# Pharmacological actions and applications of safflower flavonoids

**DOI:** 10.3389/fnut.2025.1637053

**Published:** 2025-08-06

**Authors:** Fajian Ren, Zhen Tan, Sheng Hu, Chaolong Rao, Qiwen Xiang, Jiayu Wen, Yan Chen, Cheng Peng

**Affiliations:** ^1^School of Public Health, Chengdu University of Traditional Chinese Medicine, Chengdu, China; ^2^State Key Laboratory of Traditional Chinese Medicine Resources in Southwest China, Chengdu, China

**Keywords:** *Carthamus tinctorius* L., safflower, flavonoids, pharmacological action, mechanism, applications

## Abstract

Safflower (*Carthamus tinctorius* L.), also known as Honghua, blueflower, or prickly safflower, is a medicinal herb effective in promoting blood circulation, dredging meridians, eliminating blood stasis, and relieving pain. Safflower contains complex chemical components, including flavonoids, alkaloids, organic acids, pigments, etc. Among them, flavonoids such as chalcone compounds, quercetin, rutin, and kaempferol serve as the material basis for the pharmacological effects of safflower. Flavonoids in safflower exhibit diverse biological activities, including cardiovascular and cerebrovascular protection, antioxidant, neuroprotective, antitumor, anti-inflammatory, and immunomodulatory effects. Additionally, they show great potential in gynecological diseases, food production, and other fields. With extensive research by scholars on the active mechanisms and targets of safflower flavonoids, their application prospects in the medical field have been found to be extremely broad. This review used keywords such as “*Carthamus tinctorius* L.,” “safflower,” “flavonoids,” “therapeutic effect,” “mechanism,” “application,” etc., to search relevant studies up to January 2025 in multiple internationally recognized databases (including PubMed, Web of Science, CNKI, Wan fang, Sci Finder, Elsevier, cnipa), finally including 143 high-quality studies. Representative images were drawn using BioRender and ChemDraw software to focus on presenting the action mechanisms and targets of safflower flavonoids. This article systematically reviews the pharmacological effects of safflower flavonoids and their applications in various fields in recent years, aiming to provide theoretical guidance and scientific basis for the comprehensive utilization of safflower resources and further research.

## Graphical abstract

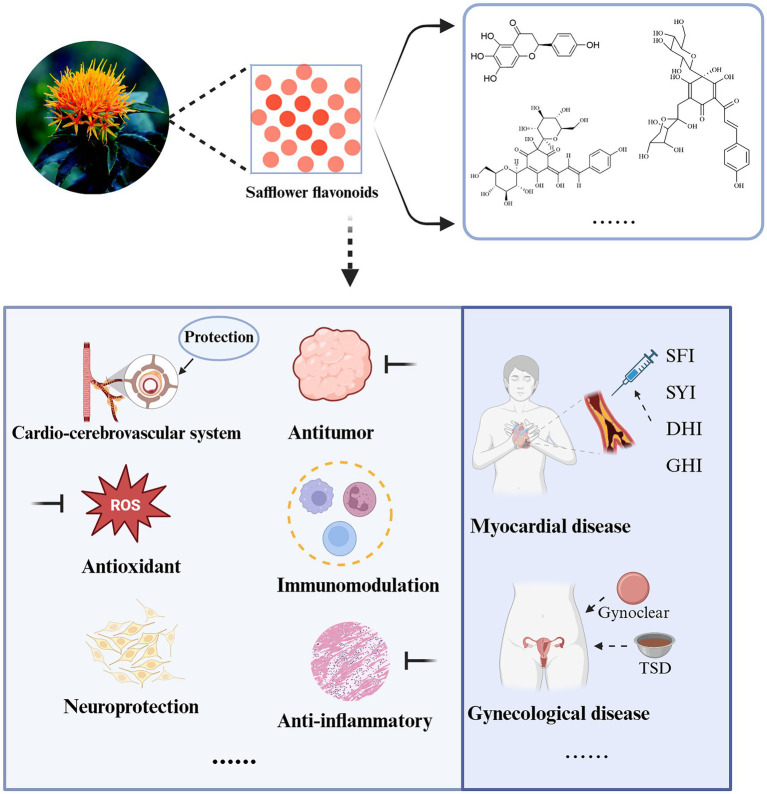

## 1 Introduction

*Carthamus tinctorius* L., known as safflower in traditional Chinese medicine, features a pungent, faintly bitter flavor and warm characteristics, associated with the heart and liver meridians. Known for its extensive biological activity, it has been used for millennia in Asia to enhance blood circulation and prevent clotting (1, 2). It is reported that safflower is cultivated in approximately 60 countries around the world (3). Current research indicates that flavonoids are the main components of safflower (4, 5). Research on the biological activity and mechanisms of safflower has been conducted by scholars globally since the last century, revealing that safflower flavonoids are beneficial to human health and have significant medical applications (6).

**Figure 1:**
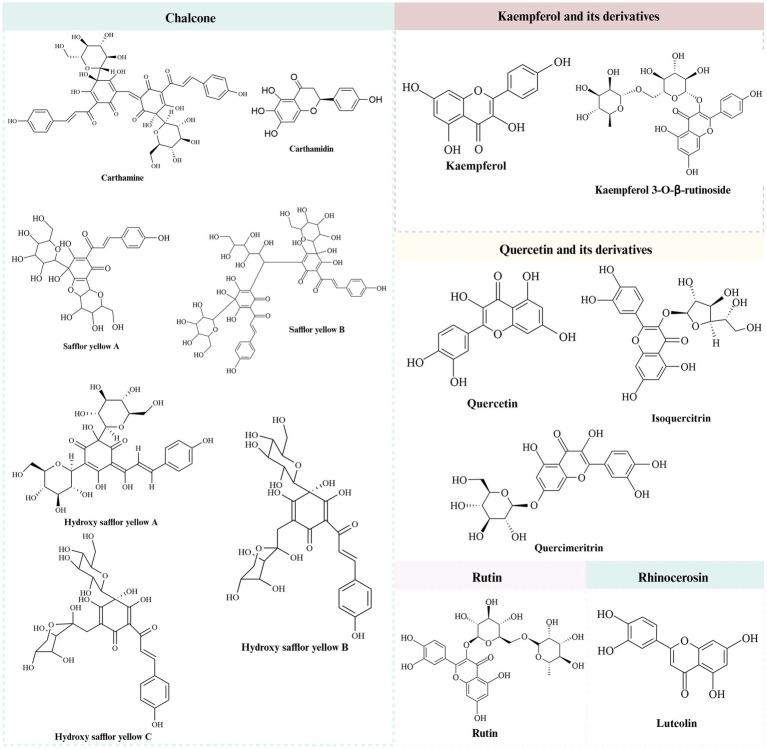
The chemical structures of safflower flavonoids.

It was found that safflower flavonoids can be categorized into chalcones, a series of derivatives with kaempferol and quercetin as the basic parent nucleus, rutin, lignans, and other compounds, depending on their structures (7). Chalcones are mostly pigmented components, divided into safflower yellow (SY) and safflower red. SY pigment is an edible natural pigment composed of various water-soluble components. After further separation, the mixture predominantly includes safflor yellow B (SYB), hydroxy safflor yellow A (HSYA), hydroxy safflor yellow B (HSYB), and additional compounds (8). HSYA, a key water-soluble quinol chalcone C-glycoside pigment and quality marker of safflower (9, 10). The specific structure is shown in Table 1 and Figure 1 (11).

**Table 1:** The chemical composition of safflower flavonoids.

Form	Ingredient	Molecular formula
Chalcone	Carthamine	C_43_H_42_O_22_
	Carthamidin	C_15_H_12_O_6_
	Safflor yellow A	C_27_H_30_O_16_
	Safflor yellow B	C_48_H_54_O_27_
	Hydroxy safflor yellow A	C_27_H_32_O_16_
	Hydroxy safflor yellow B	C_27_H_32_O_16_
	Hydroxy safflor yellow C	C_27_H_32_O_16_
Kaempferol and its derivatives	Kaempferol	C_15_H_10_O_6_
	Kaempferol 3-O-β-rutinoside	C_27_H_30_O_15_
Quercetin and its derivatives	Quercetin	C_15_H_10_O_7_
	Isoquercitrin	C_21_H_20_O_12_
	Quercimeritrin	C_21_H_30_O_16_
Rutin	Rutin	C_27_H_30_O_16_
Rhinocerosin	Luteolin	C_15_H_10_O_6_

The study concludes that safflower flavonoids exhibit diverse pharmacological effects, including cardiovascular and cerebrovascular protection, antioxidant, neuroprotective, antitumor, anti-inflammatory, and immunomodulatory activities in living organisms. The primary bioactive mechanisms are detailed in Table 2. This article systematically reviews the recent advances in research on the biological activities and action mechanisms of safflower flavonoids. Additionally, it compiles their therapeutic applications, thereby providing guidance for the future development and rational utilization of this resource.

**Table 2:** The main biological activity mechanisms of safflower flavonoids (↑increase/enhancement, ↓ decrease/inhibition).

Research target	Model	Observation condition	Mechanism of action	Bibliography
HSYA	Acute myocardial infarction in C57 mice	Intraperitoneal injection of HSYA (25 mg/kg), twice a day, for a total of 14 days	Nucleolin, VEGF-A, MMP-9 ↑	(10)
HSYA	MI mouse	Intravenous injection of HSYA (15, 30, 60 mg/kg) was administered once daily for 28 days	Bcl-2/Bax ratio, HO-1, VEGF-A, SDF-1α ↑ cleaved-caspase3, TGF-β, collagen type I levels, impaired left ventricular function ↓	(100)
HSYA	Mesenteric endothelial cell	HSYA (10^−7^, 10^−6^, 10^−5^, 10^−4^ M)	Calcium ion concentration, PKA, eNOS and its phosphorylation, NO↑	(32)
HSYA	MCAO SD rats	Intravenous injection of HSYA (10, 20, 40 mg/kg), for a total of 7 days	PGI2, cerebrovascular ↑ TXA2 ↓	(19)
HSYA	Atherosclerotic animals		PAF, TXA2, platelet aggregation ↓	(28)
HSYA	OA-induced SH-SY5Y cells	HSYA (0.2, 0.5, 1 μmol/L)	Tau protein hyperphosphorylation ↓	(135)
HSYA	Primary midbrain cells of mice	HSYA (0, 20, 40, 80, 160, 320, 640 μM), 1 h	NF-κB, iNOS, NO, IL-1β, TNF-α↓	(136)
HSYA	SCI SD Rats	After the injury, 8 mg/kg was administered 1 h and 6 h later, followed by 14 mg/kg, given once a day for a total of 7 days	TNF-α, IL-6, inflammatory mediators (iNOS and COX-2) ↓	(39)
HSYA	Rat insulinoma cell model	HSYA (800 μM)	SOD, GSH-Px, CAT↑ MDA↓ JNK/c-Jun signaling pathway↓	(33)
HSYA	HG-induced damage to HUVEC	HSYA (0–50 mM)	H_2_O_2_ and ROS↓ Activation of NOX4↓ Adhesion of adhesion molecules and monocyte EC ↓	(30)
HSYA	Rat model of DN	Gavage with HSYA (120 mg/kg), once a day, for a total of 56 days	BAX and caspase-3 ↓, BCL-2↑ TNF-α, FFA and LDH↓ SOD↑, MDA↓	(37)
HSYA	A model of H_2_O_2_-induced oxidative damage in HUVEC	HSYA (4, 8 μg/mL)	GSH/GSSG ratio, SOD↑ Akt and Bcl-2 protein ↑ ROS, BAX and PTEN protein expression ↓	(38)
HSYA	Rat TBI model	Single oral administration of HSYA (30 mg/kg)	SOD, CAT, GSH, GSH/GSSG↑ IL-1β, IL-6, TNF-α, TLR4, NF-κB↓ Bax, caspase-9, caspase-3↓	(40)
HSYA	Mouse model of HCC	Intraperitoneal injection of HSYA (2.25, 1.13, 0.57 mg/kg), twice a day, for a total of 11 days	FOXP3, RoRγt, regulatory T cells (Treg) ↓	(56)
HSYA	Hep-G2 cells	HSYA (32 μM/mL)	p62, p-ERK/ERK1/2↓ LC3-II, Beclin 1↑	(57)
HSYA	H22 liver cancer mice or Hep-G2 cells	Intraperitoneal injection of HSYA (1.125, 2.25 mg/kg) was administered once daily for 14 consecutive days HSYA (80 μM)	p38MAPK, ATF-2, COX-2, MMP-2, MMP-9↓	(137)
HSYA	LPS-induced proliferation of human non-small cell lung cancer cells A549 cells and H1299 cells	HYSA (5, 10, 20 μM)	Bcl-2, TNF-α, IL-6 and IL-1β, COX-2, MMP-2, MMP-9↓ Bax, cleaved caspase-3, cleaved caspase-9, IL-10↑ PI3K/Akt/mTOR and ERK/MAPK signaling pathways ↓	(58)
HSYA	Gastric cancer cells (BGC-823 cells)	HSYA (100 μM)	PPARγ and caspase-3↑	(59)
HSYA	Human ovarian cancer cells (Skov3 cells)	HSYA (20, 100 mg/L)	WSB1 ↓	(60)
HSYA	Endometritis in mice	Intravenous injection of HSYA (4 mg/kg)	MPO, CD45, CD3, ED-1↓ TNF-α, IL-1β, IL-6↓ p-p65/p65, p-IκBα/IκBα, p-p38/p38, p-Erk/Erk, p-JNK/JNK expression ratio↓	(71)
HSYA	Diabetic renal fibrosis in rats	Gavage with HSYA at a dose of 10 mg/kg, once daily, for a total of 42 days	IL-6, TNF-α, TLR4, NF-κB (p65) ↓	(72)
HSYA	I/R injury in rats	HSYA (4, 8, 16 mg/kg) was intravenously injected 30 min before the ischemic surgery and 30 min after the start of reperfusion	TNF-α, IL-1β, IL-18, NLRP3, ASC, caspase-1↓	(12)
HSYA	BV-2 mouse microglia	HSYA (1, 2.5, 5, 10 and 20 μM)	IL-6, iNOS↓ Arg-1, IL-4, IL-10 and IL-13↑	(75)
HSYA	Human fetal lung fibroblasts (MRC-5 cells)	HSYA (5, 15, 45 μmol/L)	IκBα↓ IKK, NF-κB (p65)↑	(74)
HSYA	Rats	Intraperitoneal injection of HSYA (16, 32, 64 mg/kg)	TNF-α↓ ICAM-1mRNA, IL-6, IL-1β↓	(138)
HSYA	Lung metastasis of hepatocellular carcinoma H22 cells in mice	Intraperitoneal injection of HSYA (0.57, 1.13, 2.25 mg/kg), twice a day, for a total of 11 days	ECM degradation, epithelial-mesenchymal transition ↓ SMMC-7721 cell proliferation, invasion and migration ↓	(62)
HSYA	Venous endothelial cells	HSYA (0.20 mmol/L)	6-keto-PGF1α, SOD↑ MDA, apoptosis rate ↓	(63)
HSYA	H9c2 cardiomyocytes	HSYA (12.5 μM) was pre-treated for 4 h	Caspase-3, NLRP3↓	(139)
HSYA	NPCM and human induced pluripotent stem cell-derived cardiomyocytes (hiPSC-CM)	HSYA (2.5, 5 and 10 μM) was pre-treated for 4 h	Cardiac enzyme levels ↓	(64)
HSYA	MCAO rat	Intravenous injection of HSYA (5 mg/kg)	mPTP opening, mitochondrial CytC output ↓	(65)
HSYA	Mouse model of polycystic ovary syndrome	Intraperitoneal injection of HSYA (3.5 mg/kg) was administered once a day for 15 consecutive days	STAR, Hsd3b1, Cyp11a1↑ Cyp19a1↓ SOD, GSH-Px, CAT, GSH, GSH/GSSG↑ MDA↓	(41)
HSYC	ICR mice	Intragastric administration of HSYC (12.5, 25, 50 mg/kg), once a day, for a total of 5 days	Thrombin and prothrombin action time, fibrinolytic system activity ↑	(26)
SYB	SD rats	For 3 consecutive days prior to I/R injury, 2 mL of SYB (6 mg/kg) was intravenously injected	cAMP, CREB, Nrf2 pathway↑ lncRNAs (AK046177), miRNAs (miR-134) ↓	(48)
SY	APP/PS1 mice	SY (10, 30, 100 mg/kg), administered once a day, for a duration of 90 days	BACE1 ↑ Aβ ↓	(140)
SY	HUVEC-12, human bone marrow mesenchymal stem cells	SY (4.5, 9, 18 μg/mL) was treated under hypoxic conditions for 12 h	HIF-1, p-VHL, VEGF ↑	(141)
SY	Cardiac ischemia/reperfusion models	10 min before LAD artery ligation, CY (10 mg/kg) was intravenously injected	Lactate dehydrogenase, ROS↓	(14)
SY	BALB/c mice	SY (5, 10 and 20 mg/kg), administered once daily, for a total of 21 days of treatment.	MMP-9, p-SRC protein ↓	(142)
SE	Mice with depression	Intragastric administration of SE (10, 30 mg/kg), once a day, for a total of 28 days	TNF-α, IL-1β, IL-6↓ TLR4, p-p38↓	(70)
HSYA, AHSYB	PC12 cell oxidative stress model	HSYA (40, 60, 80 μM), AHSYB (20, 40, 60 μM)	system xc, GPX4↑ Restoration of GSH/GSSG, ROS and iron ion levels	(24)
HSYA, AHSYB	Rat cerebral ischemia/reperfusion model	Intravenous injection of HSYA (2, 4, 8 mg/kg), AHSYB (2, 4, 8 mg/kg) was administered once a day for 3 consecutive days	GSH-Px, SOD↑ SIRT1 pathway-related mRNA and protein ↑ ROS, MDA↓	(23)
HSYA	Rats with diabetic retinopathy	Intraperitoneal injection of HSYA (50 mg/kg) was administered once daily for a total of 42 days	Bcl-2, Nrf2, HO-1↑ P53↓ Antioxidant enzymes ↑ IL-1β, TNF-α, MDA↓	(73)
SAFE	6-OHDA-induced rat model	Intragastric administration of SAFE (25, 50, 100 mg/kg), once a day, for a total of 21 days	TH, dopamine metabolism ↑ Striatal Iba-1 protein, NLRP3 inflammatory vesicles ↓	(46)
SE	Cerebral ischemia/reperfusion injury in SD rats	Intraperitoneal injection of SE at a dose of 1.25 g/kg, once a day, for a total of 12 days	GSH, Nrf2, Trx↑	(143)

6-OHDA, 6-Hydroxydopamine; Aβ, amyloid β-protein; Akt, protein kinase B; APP, amyloid precursor protein; Arg-1, arginase-1; ASC, apoptosis-associated speck-like protein containing a CARD; ATF-2, activating transcription factor-2; BACE1, beta-secretase 1; BAX, Bcl-2 associated X protein; Bcl-2, B-cell lymphoma-2; cAMP, cyclic adenosine monophosphate; CAT, catalase; COX-2, cyclooxygenase-2; CREB, CAMP-response element binding protein; CytC, cytochrome complex; DN, diabetic nephropathy; EC, endothelial cell; ECM, extracellular matrix; eNOS, endothelial nitric oxide synthase; ERK, extracellular regulated protein kinases; FFA, free fatty acids; FOXP3, forkhead box protein p3; GSH, glutathione; GSH-Px, glutathione peroxidase; GSSG, glutathione oxidized; HG, high glucose; HCC, hepatocellular carcinoma; HIF-1, hypoxia-inducible factor-1; HO-1, heme oxygenase-1; HSYA, hydroxy safflor yellow A; I/R, myocardial ischemia/reperfusion; Iba-1, ionized calcium binding adapter molecule 1; ICAM-1, intercellular cell adhesion molecule-1; IκB, inhibitor of NF-κB; IKK, inhibitor of kappa B kinase; IL, interleukin; iNOS, inducible nitric oxide synthase; JNK, c-Jun N-terminal kinase; LDH, lactate dehydrogenase; LPS, lipopolysaccharide; lncRNAs, long non-coding ribonucleic acids; MCAO, middle cerebral artery occlusion; MDA, malondialdehyde; MI, myocardial infarction; miRNAs, microRNAs; MMP, mitochondrial membrane potential; MPO, myeloperoxidase; mTOR, mammalian target of rapamycin; mPTP, mitochondrial permeability transition pore; NF-κB, nuclear factor kappa-B; NLRP3, NOD-like receptor heat protein domain associated protein 3; NO, nitric oxide; NPCM, neonatal rat primary cardiomyocytes; NOX4, NADPH oxidase 4; Nrf2, nuclear factor erythroid 2-related factor 2; OA, oleic acid; PAF, platelet-activating factor; PGI2, prostaglandin-I-2; PI3K, phosphatidylinositol 3-kinase; PKA, protein kinase A; PPARγ, peroxisome proliferator-activated receptor-γ; PS1, presenilins 1; PTEN, phosphatase and tensin homolog; p38MAPK, p38 mitogen-activated protein kinase; RORγt, retinoic acid receptor-related orphan receptor gamma-t; ROS, reactive oxygen species; SAFE, safflower flavonoid extract; SCI, spinal cord injury; SDF-1α, stromal cell-derived factor-1α; SE, safflower extract; SIRT1, silent information regulator 1; SOD, superoxide dismutase; TBI, traumatic brain injury; TGF-β, transforming growth factor-β; TH, tyrosine hydroxylase; TLR4, Toll-like receptor 4; TNF-α, tumor necrosis factor-α; Trx, thioredoxin; TXA2, thromboxane A 2; VEGF-A, vascularendothelial growth factor A; VHL, von Hippel–Lindau protein; WSB1, WD repeat and SOCS box containing 1; thromboxane A2; VEGF-A, vascular endothelial growth factor A; VHL, von Hippel–Lindau protein; WSB1, WD repeat and SOCS box containing 1; HSYA, hydroxy safflor yellow A.

## 2 Pharmacological effects of safflower and its flavonoids

### 2.1 Protective effects on cardiovascular and cerebrovascular system

Safflower flavonoids positively impact cardiovascular treatment. According to pharmacological studies, its main effects are the following four aspects: (1) Impact of intervention on myocardial ischemia/reperfusion (I/R) and myocardial infarction (MI); (2) Impact of intervention on middle cerebral artery occlusion and reperfusion (MCAO/R) and stroke; (3) Anticoagulant effect; (4) Protective effect on vascular cells.

#### 2.1.1 Myocardial ischemia-reperfusion and myocardial infarction

Safflower flavonoids can act extensively in cardiovascular diseases. Cardiac damage caused by ischemia-reperfusion presents a serious threat to cardiovascular well-being. Recent studies indicate that this condition can be mitigated through the upregulation of autophagy and the downregulation of NOD-like receptor heat protein domain associated protein 3 (NLRP3) inflammasome activity. This protective mechanism operates by suppressing the mammalian target of rapamycin (mTOR) signaling pathway while simultaneously stimulating AMP-dependent protein kinase (AMPK) activation. Such molecular interactions demonstrate promising therapeutic potential for alleviating myocardial injury following ischemic events and subsequent blood flow restoration (12). HSYA demonstrated significant cardioprotective effects in both living organisms and isolated tissue models by diminishing oxidative damage and programmed cell death. The compound also suppressed the janus kinase 2 (JAK2)/signal transducer and activator of transcription 1 (STAT1) signaling cascade, which contributed to improved outcomes following ischemia-reperfusion injury in cardiac tissue. This dual mechanism of action, reducing cellular stress while blocking pro-inflammatory pathways, resulted in enhanced myocardial preservation during ischemic events (13). Lu et al. (14) used SY to treat *in vivo* and *in vitro* models of I/R, demonstrating reduced injury by lowering lactate dehydrogenase and reactive oxygen species (ROS) release *in vitro*. Pre-reperfusion SY injection *in vivo* reduced myocardial damage, ROS production, and inflammation. Additionally, a study found that a 1:1 ratio of dashes and HSYA positively influenced cardio protection against myocardial ischemia by modulating the protein kinase B (Akt)/nuclear factor erythroid 2-related factor 2 (Nrf2)/heme oxygenase-1 (HO-1) pathway (15).

#### 2.1.2 Cerebral ischemia-reperfusion and stroke

Safflower flavonoids can act extensively in cerebrovascular diseases. The molecular mechanisms of MCAO/R injury are intricate. Yang et al. (16) has shown that HSYA effectively inhibits the activation of caspase-3, this suppression not only decreases the size of cerebral infarctions and improves neurological function but also boosts the phosphorylation of glycogen synthase kinase-3-β (GSK-3-β). Additionally, it diminishes the activity of inducible nitric oxide synthase (iNOS) and nuclear factor kappa-B (NF-κB) in the penumbra, which in turn modulates the phosphorylation of GSK-3-β. This mechanism helps to reduce inflammation and prevent cell death, effectively softening the impact of middle cerebral artery occlusion (MCAO) and its related injuries. Additionally, HSYA may inhibit JAK2-mediated signaling, activating p-JAK2/p-STAT3 expression and subsequently suppressor of cytokine signalling-3 (SOCS-3), which provides negative feedback on p-JAK2/p-STAT3, aiding HSYA’s therapeutic effects on ischemic stroke (17). Clinical trials reveal that HSYA inhibits thrombosis and reduces localized infarct size by modulating the prostaglandin-I-2 (PGI2)/thromboxane A 2 (TXA2) signaling pathway, improving blood rheology, and inhibiting platelet aggregation (18), it also dilates cerebral blood vessels, enhances cerebral vascular permeability (19) and regulates autophagy (20) to prevent ischemic stroke. Song et al. (21) established a rat model of acute permanent cerebral ischemia, administration of Anhydrosafflor yellow B (AHSYB) demonstrated notable therapeutic effects. The treatment enhanced neurological recovery, mitigated inflammation in brain tissue, and suppressed the expression of heat shock protein 60 as well as IL-6. Furthermore, AHSYB significantly alleviated damage caused by cerebral ischemia-reperfusion injury. Moreover, SY exhibited neuroprotective properties in cases of MCAO/R damage by improving neurological function, shrinking the area of brain tissue death, boosting microtubule-associated protein-2 (MAP-2) expression in the cortex, and suppressing both inflammatory responses and ferroptosis. These findings suggest SY could be a promising treatment option for ischemic stroke (22). Fangma et al. (23) explored the similarities between HSYA and AHSYB in treating cerebral ischemia/reperfusion injury. HSYA and AHSYB were administered to a hypoxia-glucose deprivation/reperfusion model, showcasing their promise as neuroprotective agents against oxygen-glucose deprivation/reoxygenation (OGD/R) and cerebral ischemia/reperfusion injury-induced neuronal injury. Ferroptosis and parthanatos are two types of programmed cell death associated with cerebral ischemia. Chen et al. (24) revealed that HSYA and AHSYB protect against cerebral ischemia-induced cell death by reducing ROS, inhibiting excessive activation of poly adenosine diphosphate-ribose polymerase-1 (PARP-1), decreasing production of PAR polymers and nuclear translocation of apoptosis inducing factor (AIF), suppressing ferroptosis and dependent cell death in PC12 cells, and alleviating oxidative stress.

#### 2.1.3 Anticoagulant

Regulation of platelet adhesion, activation and aggregation process, improvement of microcirculation, inhibition of thrombosis and anticoagulant effects are also one of the effects of safflower flavonoids on blood vessels. HSYA, safflor yellow A (SYA), and lignans in safflower extract (SE) have anti-aggregating effects. Platelets primarily function in hemostasis. Hemostasis generally involves three processes: adhesion, activation, and aggregation. Lu et al. (25) proposed that SE’s ability to activate blood circulation and alleviate blood stasis might be linked to its inhibition of platelet aggregation, as SE can inhibit the formation of procaspase activating compound 1 (PAC-1), which is involved in platelet membrane aggregation. HSYA and SYA in SE primarily inhibit platelet aggregation by targeting platelet membrane adenosine diphosphate (ADP) receptor transactivation, reducing PAC-1 glycoprotein expression, and modulating calcium activation. They also affect ADP-regulated intraplatelet cyclic adenosine monophosphate (cAMP) and TXA2 levels. HSYA mitigates phenyl hydrazine (PHZ)-induced stasis in zebrafish larvae tail veins by extending thrombin and plasminogen activity duration, enhancing fibrinolytic system function for anticoagulation and antithrombosis, and improving microcirculation (26). HSYA could reduce clotting and detoxify by improving blood flow and toxin removal (27). Platelet activating factor (PAF) is identified as a potent platelet aggregation activator. Clinical studies indicate that Dan Hong injection (DHI) significantly decreases PAF and activating factor expression *in vivo*, while also reducing TXA2 gene expression in rat models. This impedes platelet aggregation and helps prevent thrombosis, atherosclerosis, and related diseases (28).

#### 2.1.4 Vascular cell

Atherosclerosis is a progressive inflammatory disorder of the blood vessels characterized by the thickening of arterial walls and narrowing of the passageway. This condition restricts blood flow to critical organs, leading to serious complications such as heart attacks, strokes, and reduced oxygen supply to cardiac tissue (29). HSYA shows promise as a therapeutic agent for atherosclerosis by regulating endothelial function and offering vascular protection (28). HSYA was found to mitigate endothelial cell damage in human coronary arteries caused by oxidized low-density lipoproteins (LDL). The likely explanation for this phenomenon involves an increase in endothelial nitric oxide synthase (eNOS) and nitric oxide (NO) production, while concurrently reducing LDL receptor-1 levels and lactate dehydrogenase secretion (29). Chen et al. (30) has demonstrated that HSYA effectively counteracts the harmful effects of elevated glucose levels in HUVECs. This protective mechanism involves lowering hydrogen peroxide and ROS generation, suppressing NADPH oxidase 4 (NOX4) activity, and reducing the expression of adhesion molecules as well as endothelial cell attachment.

**Figure 2:**
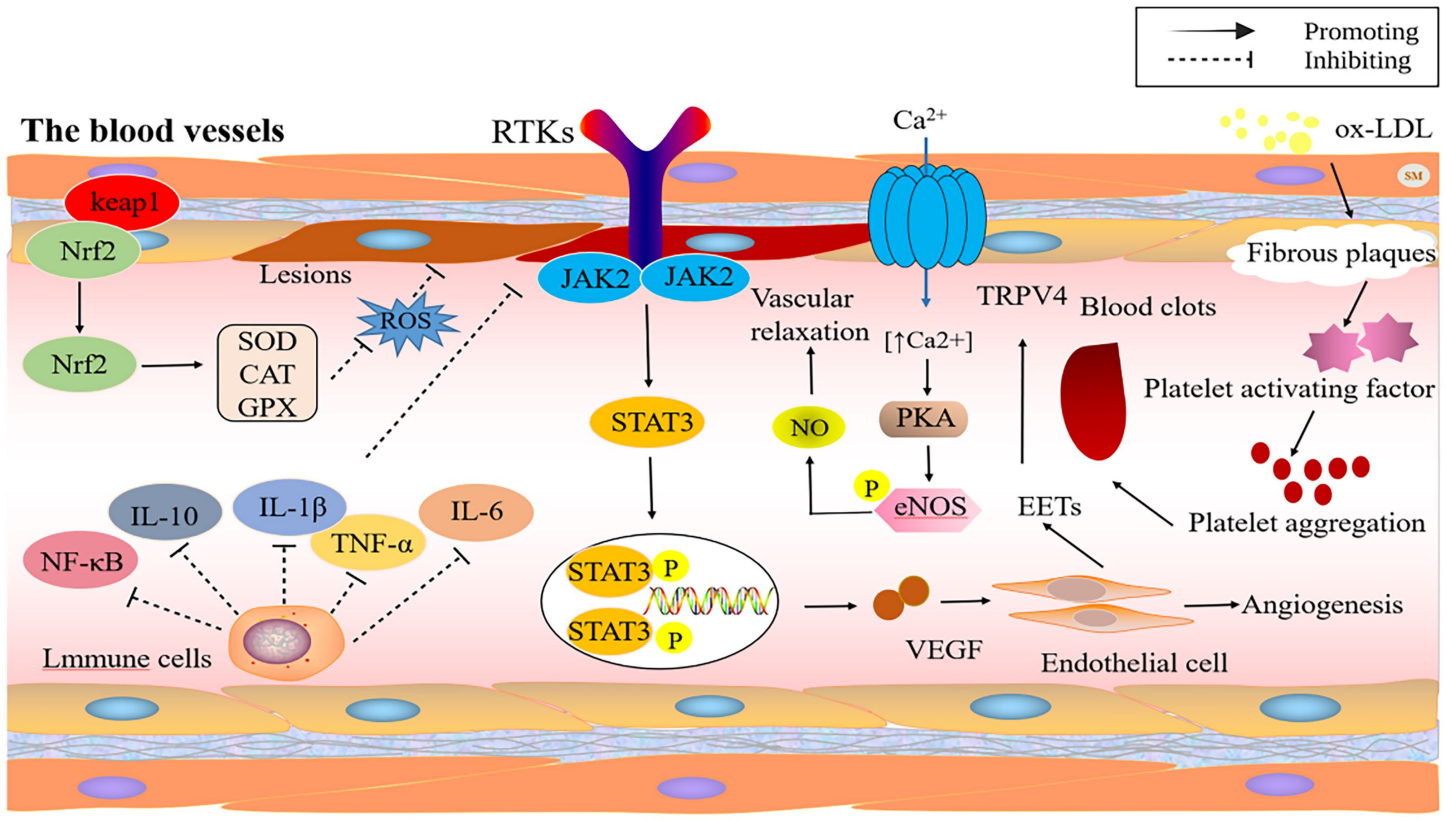
The protective mechanism of safflower flavonoids on cardiovascular and cerebrovascular system. (CAT, catalase; eNOS, endothelial nitric oxide synthase; GPX, glutathione peroxidase; IL, interleukin; JAK2, janus kinase 2; Keap1, Kelch-like ECH-associated protein 1; LDL, low-density lipoproteins; NF-κB, nuclear factor kappa-B; NO, nitric oxide; Nrf2, nuclear factor erythroid 2-related factor 2; PKA, protein kinase A; ROS, reactive oxygen species; RTKs, receptor tyrosine kinase; SOD, superoxide dismutase; STAT3, signal transducer and activator of transcription 3; TNF-α, tumor necrosis factor-α; TRPV, transient receptor potential vanilloid; VEGF, vascular endothelial growth factor).

Elevated blood pressure is similarly intimately connected with vascular cell behavior. HSYA can temper myocardial contractility by stimulating ATP-sensitive potassium channels, and big-conductance Ca^2+^ activated K^+^ channels, which consequently restore blood pressure and heart rate to normal levels in spontaneously hypertensive rats (31). Within the body’s circulatory network, the transient receptor potential vanilloid (TRPV) channels, which are found in the walls of smooth muscle, lining cells, and surrounding nerve fibers, are crucial for managing blood vessel operations and adapting to the environment. In the linings of blood vessels, when TRPV4 channels get turned on, they encourage blood vessels to relax by using pathways involving nitric oxide, prostacyclin, and potassium channels. In the smooth muscle, substances called epoxyeicosatrienoic acids (EETs) activate the TRPV4 channels, leading to the activation of potassium channels with a high conductance and resulting in the smooth muscle becoming more polarized. HSYA enhances calcium ion concentration via TRPV4 channels, activates eNOS and its phosphorylation through protein kinase A (PKA), and subsequently boosts NO production, resulting in vascular relaxation (32). Figure 2 illustrates the mechanism of action of safflower flavonoids on the cardiovascular and cerebrovascular system.

### 2.2 Anti-oxidant

Oxidative stress significantly contributes to the progression of various diseases. Safflower flavonoids possess significant antioxidant effects, this effect is mainly achieved through the following ways: (1) Inhibition of ROS and malondialdehyde (MDA), increase antioxidant enzyme activity; (2) Activation of silent information regulator 1 (SIRT1) signaling pathway; (3) Increased the expression of antioxidant factor Nrf2; (4) Regulates Bcl-2 associated X protein (BAX)/B-cell lymphoma-2 (Bcl-2) Expression, Akt/phosphatase and tensin homolog (PTEN) signaling pathway, and NF-κB; (5) Regulates glutathione (GSH) content and GSH/glutathione oxidized (GSSG).

Oxidative stress is crucial in OGD/R and ischemia/reperfusion injury, and safflower flavonoids mitigate this by enhancing antioxidant enzyme activity, reducing ROS, and modulating relevant signaling pathways. A study employing PC12 cells subjected to OGD/R as an *in vitro* oxidative stress model revealed that both HSYA and AHSYB effectively counteract cell death triggered by oxidative stress. These compounds work by enhancing the activity of the cystine/glutamate antiporter system xc and boosting glutathione peroxidase 4 (GPX4) expression. Additionally, they help rebalance the GSH/GSSG ratio, ROS, and normalize iron ion concentrations (24). SIRT1, highly expressed in the hippocampus, influences oxidative stress and apoptosis at the cellular level. Fangma et al. (23) crafted a model for cerebral ischemia/reperfusion injury, both *in vitro* and *in vivo* experiments have demonstrated that HSYA and AHSYB can exert antioxidant and anti-apoptotic effects by activating the SIRT1 signaling pathway. *In vivo*, HSYA and AHSYB boosted levels of glutathione peroxidase (GSH-Px) and superoxide dismutase (SOD), effectively mitigating oxidative stress within hippocampal neurons. *In vitro*, they discovered that HSYA and AHSYB curbed ROS and MDA output in a way that depended on the dose, while simultaneously increasing GSH-Px and SOD production. They also saw a considerable rise in mRNA and protein markers associated with the SIRT1 pathway.

Safflower flavonoids reduce high glucose-related oxidative stress by suppressing c-Jun N-terminal kinase (JNK)/c-Jun and NOX4 pathways. Zhao et al. (33) established a high glucose-induced insulinoma cell model in rats. Elevated glucose markedly raises MDA while lowering SOD, GSH-Px, and catalase (CAT), triggering JNK/c-Jun activation. The HSYA therapy effectively eases oxidative stress, bolsters antioxidant defenses, and diminishes oxidative harm and cell death in the pancreas’ beta cells. It does this by quelling the phosphorylation of JNK/c-Jun, subsequently stifling the JNK/c-Jun signaling cascade and curbing the excess production of reactive oxygen species triggered by high glucose levels. NOX4 is crucial in high glucose-induced ROS generation and apoptosis. HSYA reduces ROS levels in HUVECs via the NOX4 pathway, decreasing H_2_O_2_ and ROS production, NOX4 activation, adhesion molecules, and monocyte endothelial cell (EC) adhesion, providing an effective therapeutic effect on high glucose-induced EC injury (30).

Safflower flavonoids enhance antioxidant enzyme activity, elevate Nrf2 levels, and decrease MDA content, thereby mitigating oxidative stress associated with obesity-related diseases. Nrf2 controls cellular redox homeostasis and detoxification enzyme activation (34). Wang et al. (35) established ethanol-induced liver injury models *in vivo* and *in vitro*. Treatment with HSYA effectively reduced MDA levels, enhanced SOD and GSH levels, decreased intracellular ROS accumulation, and activated the Nrf2 pathway, demonstrating significant antioxidant effects. In a study involving rats with diabetic nephropathy, which was triggered by a high-fat diet and streptozotocin, the HSYA treatment markedly boosted SOD levels while lowering MDA levels in both the serum and kidney tissue. This suggests that HSYA has the potential to safeguard the kidneys by mitigating oxidative stress (36, 37).

Safflower flavonoids could mitigate oxidative stress through the regulation of BAX/Bcl-2 ratios, the Akt/PTEN signaling cascade, and NF-κB activation. The HUVEC oxidative damage model demonstrates that oxidative damage can lead to apoptosis. HSYA therapy boosts GSH/GSSG intracellular balance and SOD levels, reduces ROS, increases Akt and Bcl-2 protein, and diminishes BAX and PTEN protein (38). In a study involving rats with spinal cord injuries, HSYA treatment alleviated spinal cord edema, inhibited elevated MDA levels and reduced SOD activity, and significantly decreased myeloperoxidase (MPO) and nitro tyrosine levels. The underlying mechanism may involve NF-κB inhibition, which mitigates oxidative stress and prevents the release of pro-inflammatory molecules (39).

**Figure 3:**
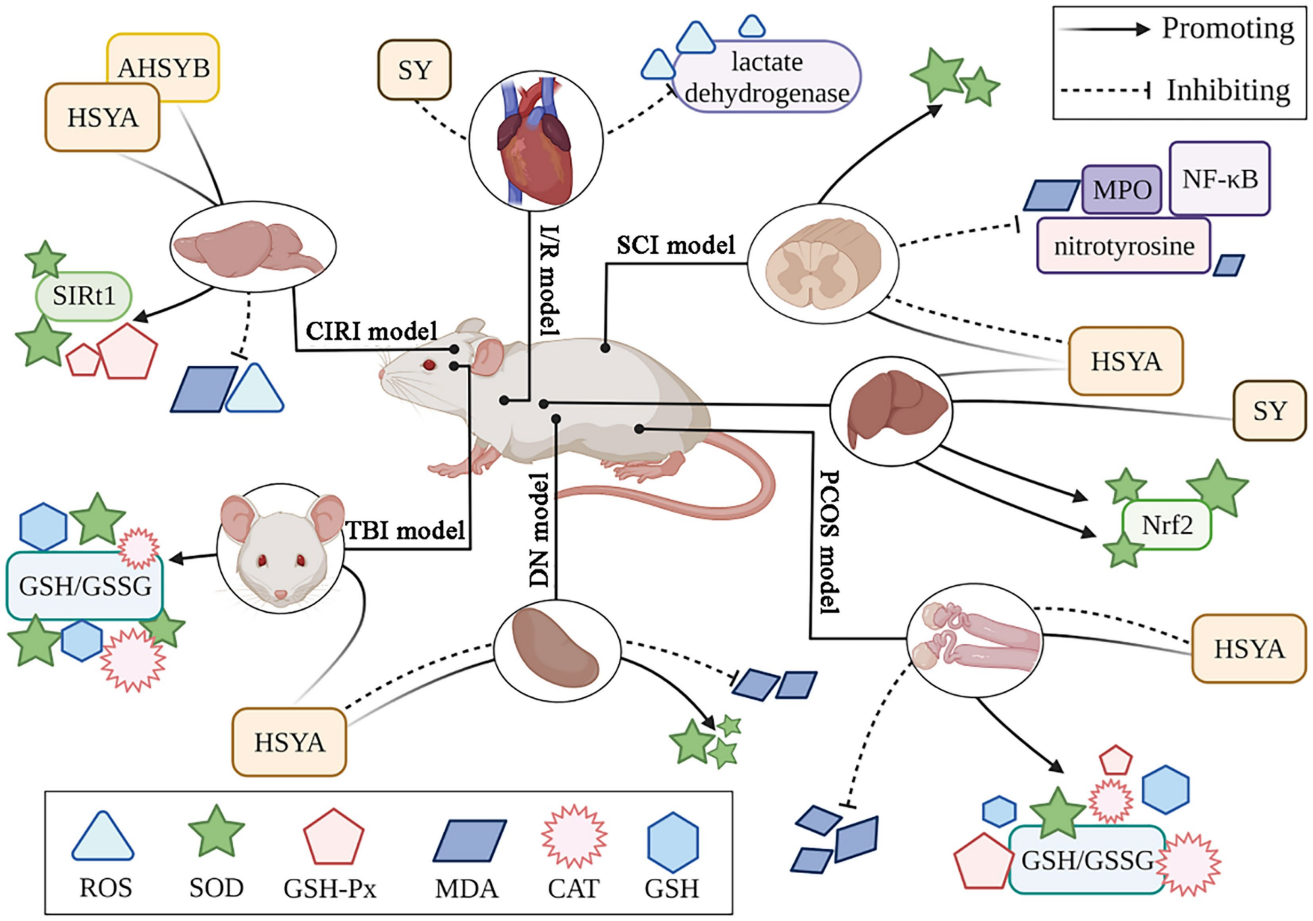
The antioxidant mechanism of safflower flavonoids. (AHSYB, anhydrosafflor yellow B; CAT, catalase; CIRI, cerebral ischemia/reperfusion injury; DN, diabetic nephropathy; GSH, glutathione; GSH-Px, glutathione peroxidase; GSSG, glutathione oxidized; HSYA, hydroxy safflor yellow A; I/R, myocardial ischemia/reperfusion; MDA, malondialdehyde; MPO, myeloperoxidase; NF-κB, nuclear factor kappa-B; Nrf2, nuclear factor erythroid 2-related factor 2; PCOS, polycystic ovary syndrome; ROS, reactive oxygen species; SCI, spinal cord injury; SIRT1, silent information regulator 1; SOD, superoxide dismutase; SY, safflower yellow; TBI, traumatic brain injury).

Safflower flavonoids have the ability to significantly enhance antioxidant enzyme activity and scavenging of MDA and ROS. Xu et al. (40) established a traumatic brain injury (TBI) model, demonstrating oxidative stress and a reduction in antioxidant enzymes post-injury. HSYA significantly increased the activity of SOD, CAT, and GSH, as well as the GSH/GSSG ratio within the damaged cortex. Luo et al. (41) established a polycystic ovary syndrome (PCOS) animal model by administering dehydroepiandrosterone. The study revealed that HSYA boosted the function of key antioxidant enzymes: SOD, GSH-Px, and CAT. Additionally, HSYA lowered MDA concentrations while increasing both GSH levels and the GSH/GSSG ratio in ovarian tissue affected by PCOS. Furthermore, HSYA and SYA significantly prolonged LDL oxidation delay time and mitigated copper-induced LDL lipid peroxidation. HSYA and SYA reduce oxidative stress in tert-butyl hydroperoxide (t-BOOH) exposed HuDe cells, mitigating ROS generation (42). Figure 3 illustrates the antioxidant mechanism of safflower flavonoids.

### 2.3 Neuroprotection

The neuroprotective effects of safflower flavonoids are demonstrated in the following neurological diseases: (1) Suppress inflammation and safeguard against spinal cord injury; (2) Improve dopamine (DA) metabolism in the substantia nigra striata pathway to protect against Parkinson’s disease (PD) neurotoxicity; (3) Control inflammation and oxidative stress, critical contributors to brain damage from ischemia-reperfusion; (4) Eliminate beta-amyloid protein deposits to slow Alzheimer’s disease (AD) progression.

Spinal cord injury (SCI) is a severe disorder with significant global health and economic consequences. Uncontrolled traumatic spinal cord injury exacerbates pathological events including increased blood-spinal cord barrier (BSCB) permeability, tissue edema, lipid peroxidation, inflammatory cytokine release, and neuronal apoptosis (43). Pei et al. (39) suggest that HSYA demonstrates therapeutic potential in animal studies modeling human spinal injuries, including fracture dislocations and burst compression fractures. Its protective effects appear to stem from reducing oxidative stress through free radical neutralization while simultaneously suppressing key inflammatory pathways, particularly by downregulating tumor necrosis factor-α (TNF-α) and blocking the expression of inflammatory enzymes like iNOS and cyclooxygenase-2 (COX-2). This dual action mechanism shows promise for mitigating neural damage in both traumatic and ischemia-related spinal cord injuries.

PD is one of the most common neurodegenerative diseases worldwide, and an increasing number of studies are focusing on the development of drugs that can effectively prevent dopaminergic cell death (44, 45). In a study employing a 6-hydroxydopamine (6-OHDA)-induced PD animal model, scientists observed notable improvements following a three-week treatment with different safflower flavonoid extract (SAFE) doses. The results showed a marked rise in spontaneous movement crossings among PD rats, alongside encouraging upward trends in tyrosine hydroxylase (TH) expression and DA metabolic activity. The nigrostriatal pathway DA is a key clinical marker for PD diagnosis. SAFE demonstrated a significant decrease in astrocyte reactivity within the substantia nigra of Parkinson’s disease model rats. It also markedly lowered ionized calcium-binding adapter molecule 1 (IBA1) protein concentrations in the striatum while effectively inhibiting the activation of the NLRP3 inflammasome pathway. Within the co-culture context, kaempferol 3-o-retinoid and AHSYB bolstered neural viability, mitigated cell death, and decreased interleukin (IL)-1β and IL-10 concentrations (46).

The NF-κB pathway drives inflammation by boosting inflammatory factor synthesis. Du et al. (47) showed that SYB significantly lowered neurological impairment metrics and infarct sizes in a dosage-related fashion within cerebral ischemia/reperfusion treatment. Furthermore, SYB suppressed the elevation of pro-inflammatory markers TNF-α, IL-6, and IL-1. However, the reoxygenation from restored blood flow can worsen tissue damage and lead to excessive ROS production, which subsequently impairs mitochondrial function. Leads to neurological damage. Studies indicate that SYB detection is facilitated by increasing intracellular cAMP levels, down-regulating long non-coding ribonucleic acids (lncRNAs) like AK046177, overexpressing microRNAs (miRNAs) such as miR-134, and triggering the CAMP-response element binding protein (CREB)/Nrf2 signaling pathway to safeguard against ischemia-reperfusion damage (48).

**Figure 4:**
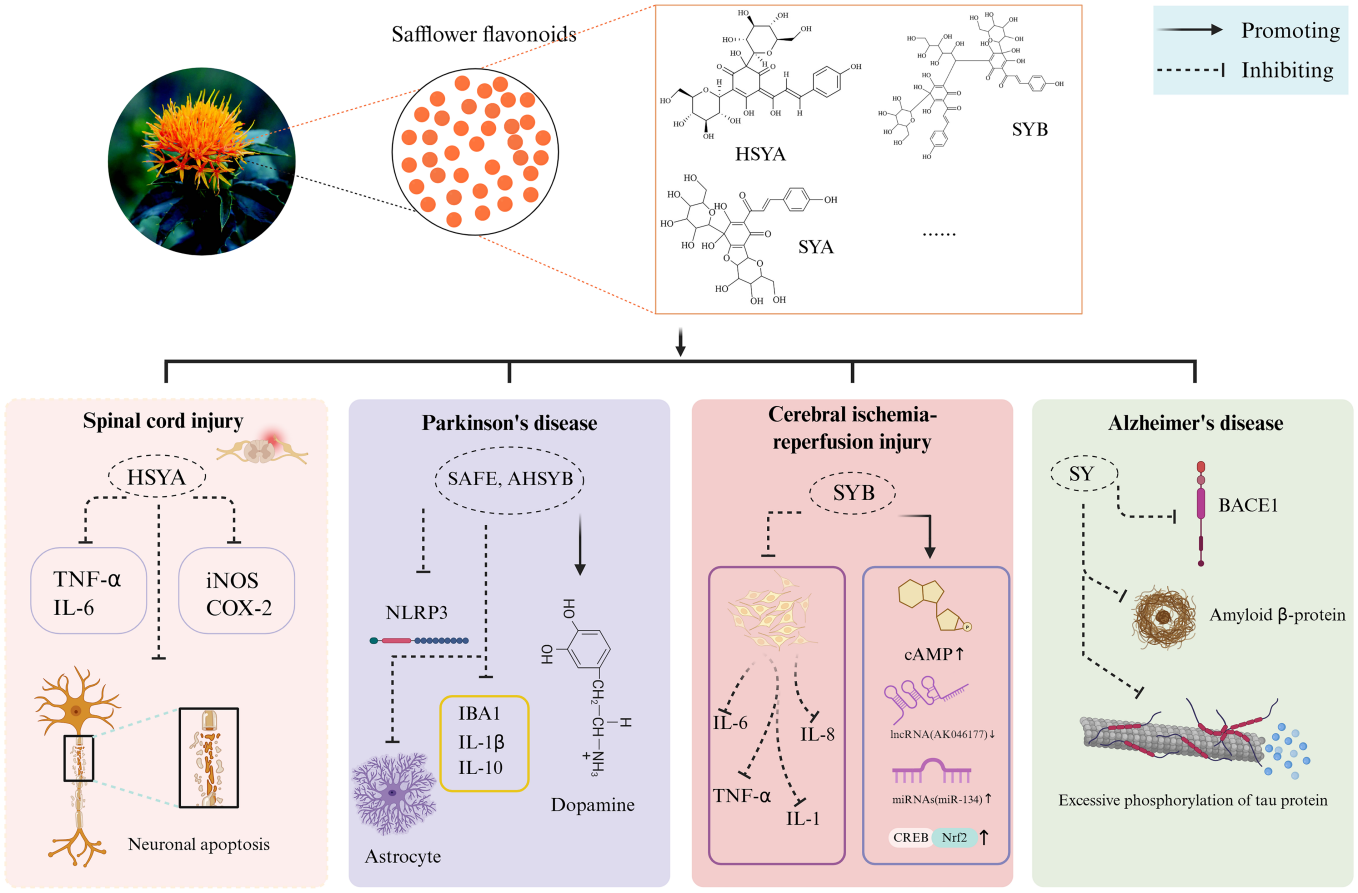
The neuroprotective mechanism of safflower flavonoids. (AHSYB, anhydrosafflor yellow B; BACE1, beta-secretase 1; cAMP, cyclic adenosine monophosphate; COX-2, cyclooxygenase-2; CREB, CAMP-response element binding protein; HSYA, hydroxy safflor yellow A; IBA1, ionized calcium-binding adapter molecule 1; IL, interleukin; iNOS, inducible nitric oxide synthase; NLRP3, NOD-like receptor heat protein domain associated protein 3; Nrf2, nuclear factor erythroid 2-related factor 2; SAFE, safflower flavonoid extract; SY, safflower yellow; SYA, safflor yellow A; SYB, safflor yellow B; TNF-α, tumor necrosis factor-α). Created in BioRender. Nû, SÑ. (2025) https://BioRender.com/rox0jvd.

AD involves severe spatial memory impairment, driven largely by Amyloid β-protein (Aβ) accumulation (49). Fluctuations in the synthesis and removal of Aβ, often triggered by inflammatory factors, result in an excessive build-up of Aβ, particularly Aβ1-42, outside the neurons. This buildup then causes harmful effects on the neurons (50). Consequently, AD patients exhibit symptoms of neurodegeneration and cognitive impairment. The formation of Aβ peptides stems from the amyloid precursor protein (APP), which typically undergoes cleavage by α-secretase and γ-secretase enzymes. This standard processing pathway not only prevents the accumulation of harmful Aβ fragments but also yields the beneficial soluble amyloid protein precursor α (sAPPα) as a byproduct. Under healthy physiological circumstances, this mechanism maintains a delicate balance that safeguards against neurotoxic buildup. Alternatively, β-secretase and γ-secretase produce neurotoxic Aβ (51). In the APP/presenilins 1 (PS1) mouse model, treatment with SY promoted APP catabolism towards sAPPα secretion in the hippocampus. Du et al. (52) shows that blocking the beta-secretase 1 (BACE1) enzyme can help clear the Aβ plaques, as thorough studies indicate that a high dosage of SY can also reduce BACE1 activity in Alzheimer’s disease mouse models. SY exhibits strong therapeutic effects by regulating secretory enzymes to suppress Aβ accumulation, providing neuroprotection against AD in APP/PS1 mice. Mevalonate pyrophosphate decarboxylase (MVD) and apolipoprotein E (APOE) regulate cholesterol synthesis, secretion, and metabolism in neurons. It has been shown that when both are expressed abnormally, it will increase the probability of AD. After administering SY orally at a dose of 30 mg/kg daily for 4 weeks to both 12-month-old APP/PS1 transgenic mice and wild-type mice exhibiting AD markers, scientists observed a marked decrease in the intensity and spread of fluorescent amyloid plaques within the cortical and hippocampal regions of the transgenic subjects. Moreover, there was a significant reduction in cholesterol, MVD, and APOE levels within the mice’s cerebral regions, hinting that minimizing the body’s own cholesterol might serve as a potent therapeutic approach against Alzheimer’s (52). The neuroprotective mechanism of safflower flavonoids is illustrated in Figure 4.

### 2.4 Anti-tumor

Flavonoids primarily combat tumors by inhibiting proliferation, inducing apoptosis, regulating angiogenesis, enhancing immune function, and modulating gene expression and signaling pathways in cancer cells (53). Safflower flavonoids exhibit the four anti-tumor mechanisms and positively impact combating breast, liver, lung, stomach, and ovarian cancers.

Safflower flavonoids positively influence breast cancer treatment. HSYB therapy inhibits the MCF-7 cell cycle at the S phase and reduces the expression of cyclin D1, cyclin E, and cyclin-dependent kinases 2 (CDK2). The compound further suppresses the expression of p-PI3K, phosphatidylinositol 3-kinase (PI3K), Akt, and p-Akt proteins. Additionally, it reduces Bcl2 concentrations while elevating Bax levels. This cascade triggers the activation of caspase-3 and caspase-9, ultimately leading to programmed cell death (54). Doxorubicin (DOX), a primary chemotherapeutic for breast cancer, when combined with HSYB, enhances treatment efficacy by significantly reducing Bcl-2 expression and upregulating caspase-9, Bax, and caspase-3. This combination also increases ROS and cytochrome C release, promoting apoptosis (55).

**Figure 5:**
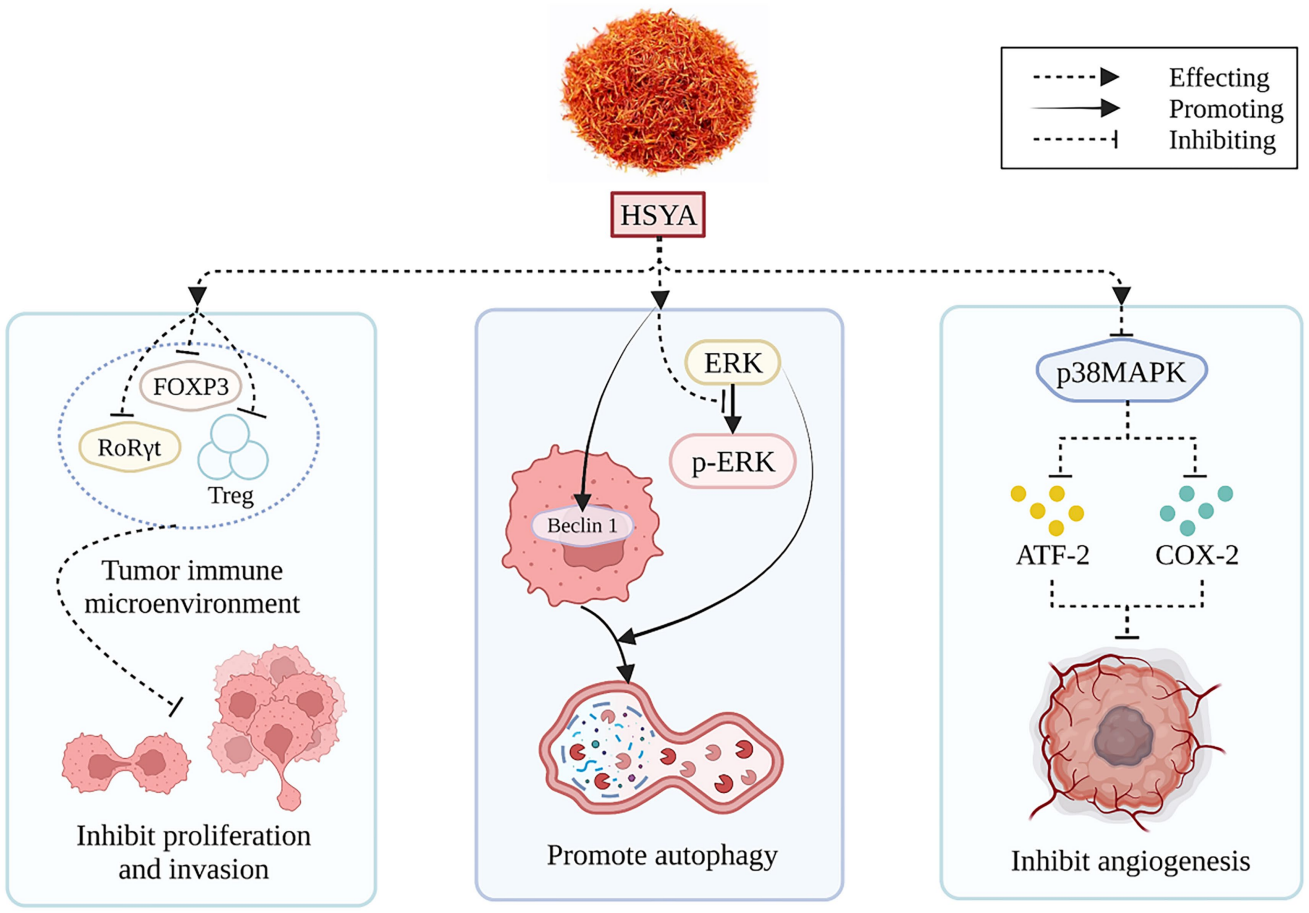
The anti-hepatocellular carcinoma mechanism of HSYA. (ATF-2, activating transcription factor-2; COX-2, cyclooxygenase-2; ERK, extracellular regulated protein kinases; FOXP3, forkhead box protein P3; HSYA, hydroxy safflor yellow A; p38MAPK, p38 mitogen-activated protein kinase; RORγt, retinoic acid receptor-related orphan receptor gamma-t; Treg, regulatory T cells).

HSYA, derived from safflower flavonoids, may positively impact hepatocellular carcinoma treatment. The immune landscape within tumors critically influences cancer cell growth and spread. In a recent investigation involving a rodent model of liver cancer, the HSYA treatment proved effective in diminishing the levels of crucial immune environment regulators, such as forkhead box protein P3 (FOXP3) and retinoic acid receptor-related orphan receptor gamma-t (RoRγt) proteins, and lowering the number of regulatory T cells in the spleen at certain dosages (56). HSYA treatment of Hep-G2 cells enhanced Beclin 1 gene expression and inhibited extracellular regulated protein kinases (ERK) phosphorylation, thereby inducing autophagy and reducing hepatocellular carcinoma cell viability (57). Figure 5 illustrates the mechanism of HSYA against hepatocellular carcinoma.

Safflower flavonoids may positively impact the treatment of lung, stomach, and ovarian cancers. Lipopolysaccharide (LPS) promoted the expansion and colony genesis in A549 and H1299 cells, whereas HSYA mitigated this action. Specifically, HSYA down-regulated Bcl-2 expression and increased levels of cleaved caspase-3, cleaved caspase-9, and Bax, promoting apoptosis. It also inhibited IL-6, IL-1β, and TNF-α production while increasing IL-10 levels. Additionally, HSYA reduced COX-2 expression, suppressed inflammatory factor release, and consequently inhibited tumor neovascularization and metastasis. It also suppressed mitochondrial membrane potential (MMP)-2 and MMP-9 expression, both associated with tumor infiltration, thus blocking LPS-stimulated movement and penetration in A549 and H1299 cells (58). HSYA treatment increased peroxisome proliferator-activated receptor-γ (PPARγ) and caspase-3 expression in BGC-823 gastric cancer cells, with its inhibitory, apoptotic, and cycle-blocking effects reliant on PPARγ activation (59). In Skov3 ovarian cancer cells, HSYA dose-dependently inhibited proliferation, reduced WD repeat and SOCS box containing 1 (WSB1) gene expression, and enhanced apoptosis, thereby suppressing neuroblastoma and ovarian cancer cell growth (60).

### 2.5 Immunomodulation

**Figure 6:**
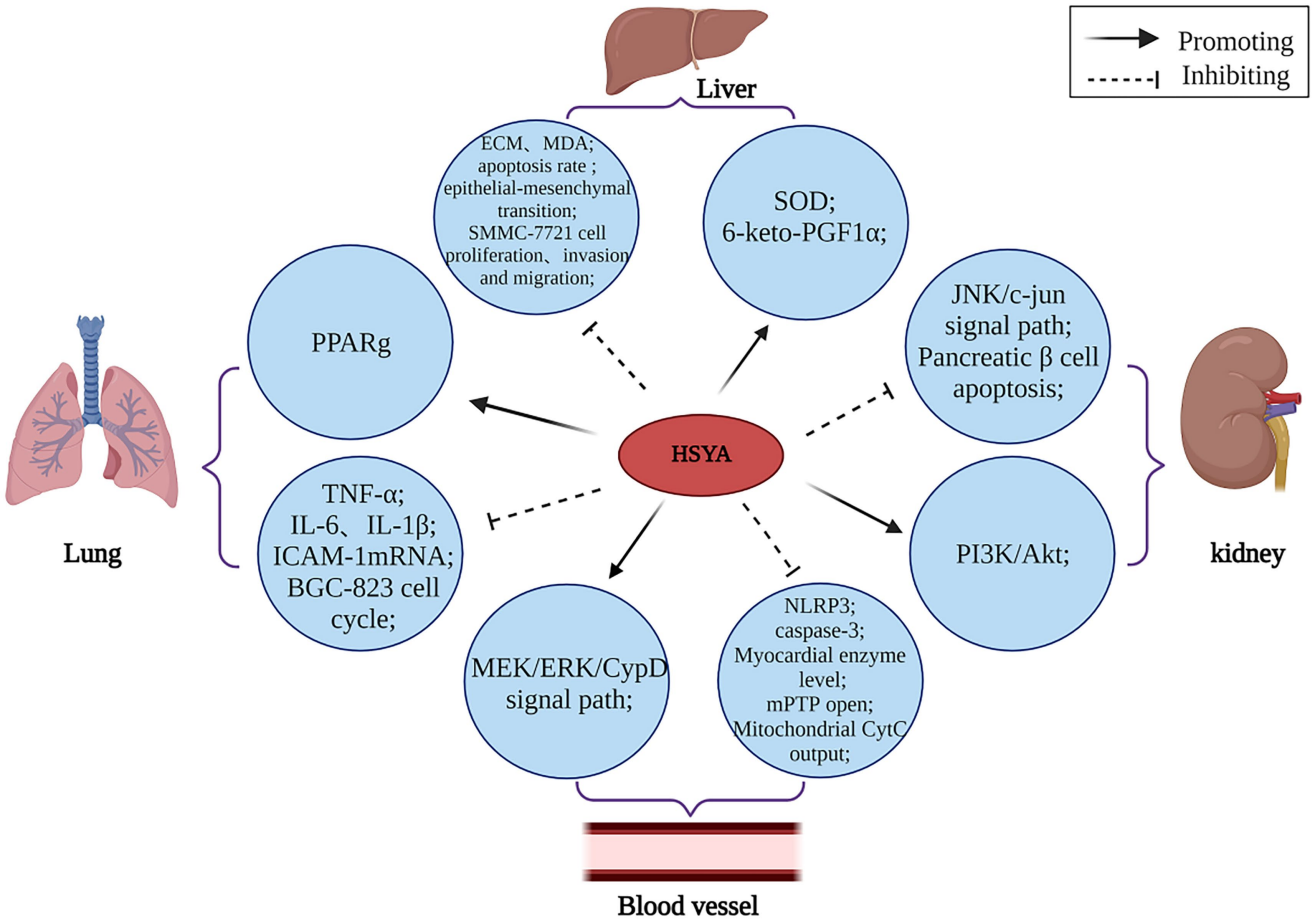
The immunomodulatory mechanism of HSYA. (Akt, protein kinase B; CypD, cyclophilin D; CytC, cytochrome complex; ECM, extracellular matrix; ERK, extracellular regulated protein kinases; ICAM-1, intercellular cell adhesion molecule-1; IL, interleukin; JNK, c-Jun N-terminal kinase; MDA, malondialdehyde; MEK, mitogen-activated extracellular signal-regulated kinase; mPTP, mitochondrial permeability transition pore; NLRP3, NOD-like receptor heat protein domain associated protein 3; PI3K, phosphatidylinositol 3-kinase; PPARg, peroxisome proliferator-activated receptor-γ; SOD, superoxide dismutase; TNF-α, tumor necrosis factor-α).

Numerous studies have established HSYA’s influence on cell proliferation, apoptosis, and pyroptosis, supporting its potential in preventing and treating various conditions. The mechanism of HSYA immunomodulatory action is shown in Figure 6.

HSYA has been demonstrated to regulate various apoptotic factors linked to PPARγ. HSYA hinders the growth of BGC-823 cells by causing PPARγ-mediated cell cycle pause and programmed cell death, suggesting PPARγ’s involvement in the induction of apoptosis by HSYA in these cells (59). HSYA helps mitigate the harshness of lung injury by cutting down on fluid buildup in the lungs, lowering the levels of acid in the blood, and dampening down inflammation. Additionally, it boosts the levels of oxygen in the blood and keeps key inflammatory proteins under control in cases of acute lung injury, such as TNF-α and intercellular adhesion molecule-1 mRNA (61). Additionally, HSYA impedes SMMC-7721 cell proliferation and adhesion to the extracellular matrix (ECM), suggesting its potential in preventing hepatocellular carcinoma lung metastasis (62). DHI, whose main active component is HSYA, plays a protective role in vascular endothelial cells during hypoxic conditions. It achieves this by increasing the concentration of 6-keto prostaglandin F1α while boosting SOD activity. Additionally, DHI helps balance the activity of plasminogen activator inhibitor (PAI) and tissue-type plasminogen activator (t-PA), as well as regulating endothelin (ET) and NO levels. These combined effects promote vasodilation and preserve normal endothelial function, effectively shielding blood vessels from hypoxia-induced damage (63).

I/R injury remains a pressing worldwide health concern. HSYA enhances cardiac contractility and restores normal field potential patterns in human-induced pluripotent stem cell-derived cardiomyocytes following hypoxia/reoxygenation injury, indicating its therapeutic potential for I/R damage. This compound demonstrates promising cardioprotective effects by addressing both mechanical and electrical dysfunction in compromised heart cells (64). Additionally, HSYA protects against ischemia/reperfusion by inhibiting the mitochondrial permeability transition pore (mPTP) via the mitogen-activated extracellular signal-regulated kinase (MEK)/ERK/Cyclophilin D (CypD) pathway. This research (65) reveals a new pathway for HSYA’s protection against ischemia/reperfusion injury and provides a blueprint for developing mPTP-targeting therapies.

Type II diabetes mellitus (T2DM) and its complications [diabetic nephropathy (DN), etc.] is a complex metabolic disease and a significant global public health issue. HSYA could potentially amplify PI3K/Akt signaling and hinder β-cell apoptosis in diabetic rats, thereby potentially alleviating insulin resistance and modulating glucose-lipid homeostasis (66), HSYA’s participation in the JNK/c-Jun signaling cascade hints at its potential to alleviate oxidative stress triggered by high glucose levels. This discovery could be a game-changer in the fight against diabetes, both in terms of prevention and therapeutic approaches (33).

### 2.6 Anti-inflammatory

Safflower contains flavonoids known for their anti-inflammatory properties (67). The anti-inflammatory effects of saffower largely from HSYA, acting through: (1) inhibiting the toll-like receptor 4 (TLR4)/NF-κB pathway; (2) lowering inflammatory mediators such as IL-1β, IL-6, and TNF-α; (3) elevating anti-inflammatory mediators like IL-13, IL-4, and IL-10, thus acting via supplementary pathways.

The transcription factor NF-κB plays a pivotal role in modulating gene expression, particularly in driving pro-inflammatory pathways. According to Lee et al. (68), safflower’s ethanolic extract is a hero in the fight against oxidative stress, as it cleverly adjusts the ROS/NF-κB dance and the Nrf2/HO-1 duet to safeguard HUVECs. Yin et al. (69) suggest a link between depression and inflammation. Chen et al. (70) crafted a chronic, inconsistent, and moderate stress model in mice to study depression. They discovered that a certain enzyme, SE, plays a crucial role in regulating the TLR4-NLRP3 signaling route. It decreases the levels of TLR4, p38, and the phosphorylated form of NF-κB, which in turn stifles neuroinflammation and eases the symptoms of depression.

HSYA demonstrated a notable ability to suppress the production of key pro-inflammatory mediators. In their study, He et al. (71) examined how HSYA influenced *Staphylococcus aureus*, triggered endometrial inflammation in murine models, observing a significant reduction in TNF-α, IL-1β, and IL-6 concentrations within the affected uterine tissues. HSYA has shown promise in addressing renal fibrosis linked to diabetes in rodent studies. By quelling pro-inflammatory agents like IL-6, TNF-α, TLR4, and NF-κB (p65), it helps mitigate the signs of renal fibrosis (72). Ye et al. (12) developed a rat model of myocardial ischemia/reperfusion treated with HSYA. Test results indicated that HSYA decreased IL-1β and IL-18 release and significantly down-regulated NLRP3, ASC, and caspase-1 expression. HSYA promotes myocardial autophagy through the AMPK/mTOR axis, suppresses NLRP3 inflammasome initiation, and reduces the production of inflammatory markers to protect ischemic heart muscle. Sun et al. (73) revealed that HSYA effectively reduced retinal ganglion cell death in rats with T2DM. This protective effect was achieved by suppressing inflammatory markers like IL-1β and TNF-α while simultaneously boosting the production of key proteins, including Nrf2, HO-1, Bcl-2, and P53. Their findings highlight HSYA’s potential as a neuroprotective agent in diabetic retinopathy.

HSYA can suppress the inflammatory response by competing with pro-inflammatory factor receptors. Liu et al. (74) investigated the inflammatory response in MRC-5 cells induced by TNF-α. Their findings revealed that HSYA treatment allows HSYA to compete with TNF-α for TNF receptor type 1 (TNFR1) binding, thereby obstructing the interaction between TNFR1 and the transforming growth factor-β-activated kinase 1 (TAK1)-TGF-beta activated kinase 1 (map3k7) binding protein 2 (TAB2) complex. HSYA effectively curbed TNF-α-triggered cell proliferation and inflammatory responses in MRC-5 cells by disrupting NF-κB signaling pathways. The compound also blocked activator protein-1 (AP-1) from binding to the TGF-β1 promoter, thereby preventing the cascade of proliferative and pro-inflammatory effects typically induced by TNF-α stimulation. This dual mechanism of action demonstrates HSYA’s potent anti-proliferative and anti-inflammatory properties in this cellular model.

HSYA also exerts anti-inflammatory effects through multiple synergistic pathways. Ren et al. (75) established a chronic neuroinflammation model using Aβ1-42-induced BV-2 microglia and found that HSYA mitigates neuroinflammation via triggering receptor expressed on myeloid cells 2 (TREM2): (1) HSYA significantly downregulates the expression of pro-inflammatory factors IL-1β and IL-6, while upregulating anti-inflammatory factors IL-4, IL-10, and IL-13 via TREM2, thus alleviating neuroinflammation. (2) HSYA modulates Aβ1-42-induced microglia via TREM2, inducing their polarization from pro-inflammatory M1 phenotype (releasing abundant cytotoxic inflammatory mediators) (76) to anti-inflammatory M2 phenotype (increasing expression of anti-inflammatory mediators) (77). (3) HSYA regulates the Aβ1-42-induced TLR4/NF-κB pathway via TREM2, blocking signal transduction of the pathway, significantly downregulating TLR4 expression, and reducing the phosphorylation levels of downstream NF-κB and inhibitor of NF-κB (IκB), thereby exerting neuroprotective effects.

HSYA also inhibits inflammatory responses by competing for pro-inflammatory factor receptors. Liu et al. (74) used TNF-α-induced MRC-5 cells to establish an inflammatory response model. After HSYA treatment, it was found that HSYA could compete with TNF-α for the binding of TNFR1 and block the binding of TNFR1 to TAK1-TAB2 complex. Inhibit the proliferation and inflammatory response of MRC-5 cells induced by TNF-α. At the same time, HSYA can inhibit the activation of NF-κB signaling pathway and the binding of TGF-β1 promoter to AP-1, thereby inhibiting the proliferation and inflammatory response of MRC-5 cells induced by TNF-α.

### 2.7 Regulation of glucose metabolism

SY or HSYA can improve glucose metabolism and aids in metabolic control (1, 30). HSYA exhibits significant hypoglycemic effects, particularly at higher doses (120 mg/kg), and is safe for both injection and oral administration (37), this indicates that safflower flavonoids have broad application prospects in regulating glucose metabolism, particularly in diabetes and related diseases.

HSYA shows significant promise for diabetes care. Studies confirm its safety and efficacy for managing early stage T2DM (78, 79). Furthermore, HSYA demonstrated protective effects against glucotoxicity-induced oxidative stress and pancreatic β-cell apoptosis by mitigating oxidative damage and modulating the JNK/c-Jun signaling pathway, supporting its potential use in diabetes prevention and treatment. Zhang et al. (80) indicates that HSYA’s antidiabetic effects are primarily mediated by its antioxidant and anti-inflammatory actions, involving the JNK/c-Jun and NOX4 pathways, as well as macrophage differentiation, as demonstrated in both *in vitro* and *in vivo* studies. Yao et al. (81) showed that administering HSYA to streptozotocin-induced diabetic cardiomyopathy (DCM) mice alleviated DCM symptoms through oxidative stress reduction. Mounting evidence highlights the pivotal role of vascular inflammation in driving diabetes complications. Chronically elevated blood glucose wreaks havoc on blood vessels, but promising research suggests HSYA may offer therapeutic benefits by counteracting this vascular deterioration (28, 82).

HSYA may effectively treat persistent diabetic foot ulcers. An *in vivo* diabetic rat wound model showed that HSYA significantly enhanced vascular endothelial growth factor (VEGF) levels, promoted epidermal cell migration for re-epithelialization, and inhibited NO production dose-dependently. This accelerated wound recovery, improved healing quality, and increased TGF-β1 content, aiding epidermal barrier restoration and infection prevention. Furthermore, an *in vitro* model using HUVECs demonstrated that HSYA significantly enhances neovascularization and keratinocyte migration (83).

In addition, HSYA improves insulin resistance and SY improves insulin sensitivity, which has a positive effect on diabetes. HSYA mitigates fat accumulation, restores glycemic balance, enhances insulin sensitivity, and reduces inflammation (84). Moreover, SY enhances PPARγ expression by stimulating its promoter activity, which in turn boosts the transcription of genes linked to insulin signaling in subcutaneous fat. This mechanism leads to reduced fat accumulation, lower blood sugar levels, and enhanced insulin responsiveness in mice with diet-induced obesity (85).

### 2.8 Liver and lung protection

Numerous studies have found that safflower has liver and lung protective effects, which is why it is widely used in clinical applications (86–88).

Ao et al. (1) indicates a strong correlation between HSYA’s hepatoprotective properties and its ability to combat liver fibrosis and act as an antioxidant. HSYA has an effect on liver fibrosis, liver ischemia and other impaired liver functions. Histological studies have shown that alcohol-induced liver damage, such as liver fibrosis, is significantly reduced by HSYA. HSYA effectively lowered overall cholesterol and triglyceride concentrations in the bloodstream while also inhibiting the activation of mitogen-activated protein kinase (MAPK). Additionally, it downregulated the production of transforming growth factor receptor types I and II in rats with liver fibrosis (89, 90). Wang’s et al. (91) research has demonstrated that the quercetin and kaempferol present in safflower can alleviate hepatic steatosis, improve liver function, reduce inflammation and oxidative stress responses by upregulating the expression of NR1H4.

Hepatocellular carcinoma (HCC) ranks among the most prevalent digestive system cancers. Ma et al. (56) indicates that 1.13 mg/kg HSYA can modulate the immune microenvironment in liver cancer model mice by decreasing splenic Treg cells and the expression of FOXP3 and RORγt in tumor tissues, thereby enhancing immunity and mitigating cisplatin side effects, contributing to its anticancer effects. Additionally, HSYA could suppress tumor growth and trigger cell death by blocking autophagy in liver cancer, indicating its therapeutic promise (92).

All existing studies have shown that HSYA plays a crucial role in safeguarding the lungs, especially for individuals suffering from chronic obstructive pulmonary disease (COPD), acute lung injury (ALI), and lung fibrosis. Ling et al. (93) and Wang et al. (87) has identified SYA, HSYA, and AHSYB, the key bioactive compounds in safflower, as effective agents in reducing lung damage triggered by lipopolysaccharide and reticulation. Safflower’s ability to improve microcirculation and combat inflammation likely plays a pivotal role in easing sepsis-related lung inflammation. HSYA is a potential pulmonary vasorelaxant for treating pulmonary arterial hypertension (PAH) and related diseases. Rather than targeting endothelial cells in the pulmonary artery, this compound works by improving the function of pulmonary vascular smooth muscle. It achieves this by activating Kv channels specifically within pulmonary vascular smooth muscle cells, highlighting its promising therapeutic potential for PAH (94). Additionally, HSYA significantly inhibits airway thickening and collagen deposition in smoking- or LPS-induced rat models of COPD, reducing transforming growth factor-β1 mRNA and protein expression. This treatment also decreases airway hyperresponsiveness in asthmatic mice. HSYA injection was shown to mitigate lung histopathological changes in rats with pulmonary fibrosis, enhance body weight and oxygen partial pressure (PaO_2_), and decrease CO_2_ partial pressure (PaCO_2_), TNF-α, IL-1β, TGF-β1 mRNA expression, IL-6, MDA activity, and NF-κB p65-positive cell counts, thereby providing a protective effect on lung tissue (95).

## 3 Application potential of safflower and its flavonoids

### 3.1 Cardiovascular and cerebrovascular diseases

Cardiovascular diseases (CVD) encompass conditions affecting the heart vessels, such as acute coronary syndrome and angina, as well as cerebral vessels, including stroke, cerebral embolism, and hemorrhage. The incidence of CVD is increasing globally, so the search for new promising treatment options is becoming an urgent issue. Recently, there has been a growing focus on clinical herbal medicine in drug development. Chinese medicine is increasingly favored for disease treatment due to its minimal toxicity and side effects (96). HSYA, sourced from the therapeutic plant safflower, has shown therapeutic benefits for cardiovascular conditions, malignancies, and diabetes (97). Thus, Meng et al. (98) emphasize HSYA’s key biological effects, such as anti-inflammatory and antioxidant capabilities, along with its cardiovascular protection. Consequently, HSYA shows promising potential for preventing ischemia/reperfusion injury and treating cardiovascular diseases, as detailed in Supplementary Table 1.

Safflower flavonoids can decrease myocardial infarction size and alleviate cardiac damage due to their anti-inflammatory properties. HSYA demonstrates strong potential as a therapeutic agent for cardiovascular conditions, especially myocardial ischemia and infarction (99). HSYA enhances ischemic cardiogenic flow dynamics, boosts survival rates in mice, and mitigates ischemic cardiac dysfunction in acute MI models (10). It mitigates myocardial apoptosis and fibrosis, enhances endothelial progenitor cell activity *in vitro*, and eases cardiac injury in myocardial infarction models (100). HSYA serves as a non-toxic alternative for modulating mPTP to treat hypoxia/reoxygenation-induced injuries, protecting cardiomyocytes from pore damage (65).

Zhang et al. (80) indicates that HSYA’s pharmacokinetic properties support its extensive clinical use as an injectable for treating microvascular complications. HSYA is expected to emerge as an oral medication offering improved absorption and anti-inflammatory effects through advanced delivery technologies. Moreover, studies conducted by Yu et al. (101) underscore HSYA’s neuroprotective effects, demonstrating its ability to preserve cognitive abilities and maintain synaptic flexibility after cerebral ischemia-reperfusion damage. These findings point to HSYA’s therapeutic potential in aiding mental recovery following such injuries.

Stroke is the world’s second-highest cause of mortality and a primary cause of impairments (102), with ischemic stroke being the most prevalent subtype and a significant cause of mortality (19). Since 2005, the State Food and Drug Administration of China has approved HSYA for clinical use in treating acute myocardial infarction, angina pectoris, and other cardiac ischemia, primarily due to its antiplatelet aggregation properties (Drug Licence Document Z20050146) (103). At present, HSYA has not received marketing authorization from the U.S. Food and Drug Administration and the European Medicines Agency. HSYA shows promise in combating atherosclerosis by stifling the development of foam cells, stimulating the growth and movement of vascular smooth muscle cells, and rectifying the dysfunction of vascular endothelial cells (28). Related products include safflower injection (SFI), safflower yellow injection (SYI), DHI, and Guhong injection (GHI). A meta-analysis by Lu et al. (104) indicates that integrating SFI with conventional medication may enhance the efficacy and safety of treatment for acute coronary syndrome (ACS) patients. Li et al. (105) demonstrated that SYI combined therapy is more efficient, safer, and cost-effective, with fewer adverse effects compared to *Panax ginseng* injection. Xuan et al. (106) found that combining SYI with conventional therapy significantly improved therapeutic efficacy and cost-effectiveness in treating Chinese patients with stable angina pectoris compared to conventional therapy alone. Furthermore, DHI reduces blood–brain barrier impairment caused by mannitol (107). Widely employed in China for managing coronary heart disease, angina, myocardial infarction, pulmonary hypertension, and ischemic stroke (108, 109). Additionally, utilizing a warm microemulsion technique, Zhao et al. (110) developed HSYA solid lipid nanoparticles (SLN) with a water-in-oil emulsion structure to improve HSYA’s oral absorption and pharmacological efficacy. Moreover, the study by Gao et al. (83) demonstrated, through *in vitro* experiments, that HSYA dramatically spurred the development of new blood vessels and the movement of keratinocytes within HUVECs. Applying HSYA locally has been found to notably expedite the healing process in living organisms. Consequently, HSYA offers a viable treatment option for persistent non-healing conditions.

### 3.2 Gynecologic diseases

Safflower flavonoids exhibit significant antioxidant and anti-inflammatory properties, along with other physiological activities. Safflower is extensively utilized for treating gynecological disorders such as PCOS, endometriosis, dysmenorrhea, and irregular menstruation (6), as demonstrated in Supplementary Table 2.

HSYA was able to treat PCOS by reducing ovarian cysts and restoring normal ovulatory cycles in mice. HSYA modulates the expression of essential genes involved in ovarian steroid hormone synthesis. HSYA demonstrates antioxidant properties in PCOS ovaries by lowering MDA levels and boosting antioxidant defenses (41). HSYA has been shown to mitigate oxidative stress and reduce ROS levels, thereby inhibiting the inflammatory response in the peritoneal cavity and effectively treating endometriosis (111). Taohong Siwu Decoction (TSD), a Chinese herbal formula comprising safflower, peach kernel, angelica sinensis, chuanxiong, white peony, and ripened dihuang, is effective in treating irregular menstruation, dysmenorrhea, and gynecological inflammation. Some studies have proved that the safflower flavonoid components, kaempferol and quercetin, are the main compounds in the treatment of primary dysmenorrhea in safflower four-substance soup. Furthermore, the HSYA aspect of TSD has demonstrated its efficacy in alleviating the inflammatory reactions linked to irregular menstrual bleeding (39).

### 3.3 Food and feed applications

Safflower is also potentially important for food production and can make meals nutritious (112). Due to the occurrence of food safety incidents such as Sudan red and malachite green, people are therefore paying more and more attention to the development and application of natural colors. Research indicates that safflower pigments are commonly utilized in various food items, including meat products (113), cake toppings (9), desserts, jellies, confectionery (114), etc. Machewad et al. (115) assessed SY in’s effectiveness as an ice cream colorant, the results show that the addition of SY (0.06 mL) in the ice cream can achieve the best sensory acceptance. Variations in this amount negatively impacted flavor, color, and texture evaluations. Furthermore, research by Hong et al. (116) demonstrated that compared to plain yogurt, yogurt supplemented with SE exhibited a significant increase in total phenolic and flavonoid contents. This enhancement led to improved antioxidant activity without negatively affecting the yogurt’s characteristics. Additionally, this modified yogurt showed inhibitory effects on key enzymes involved in the digestion and absorption of carbohydrates and lipids, playing a positive role in the regulation of hyperglycemia and hyperlipidemia.

**Figure 7:**
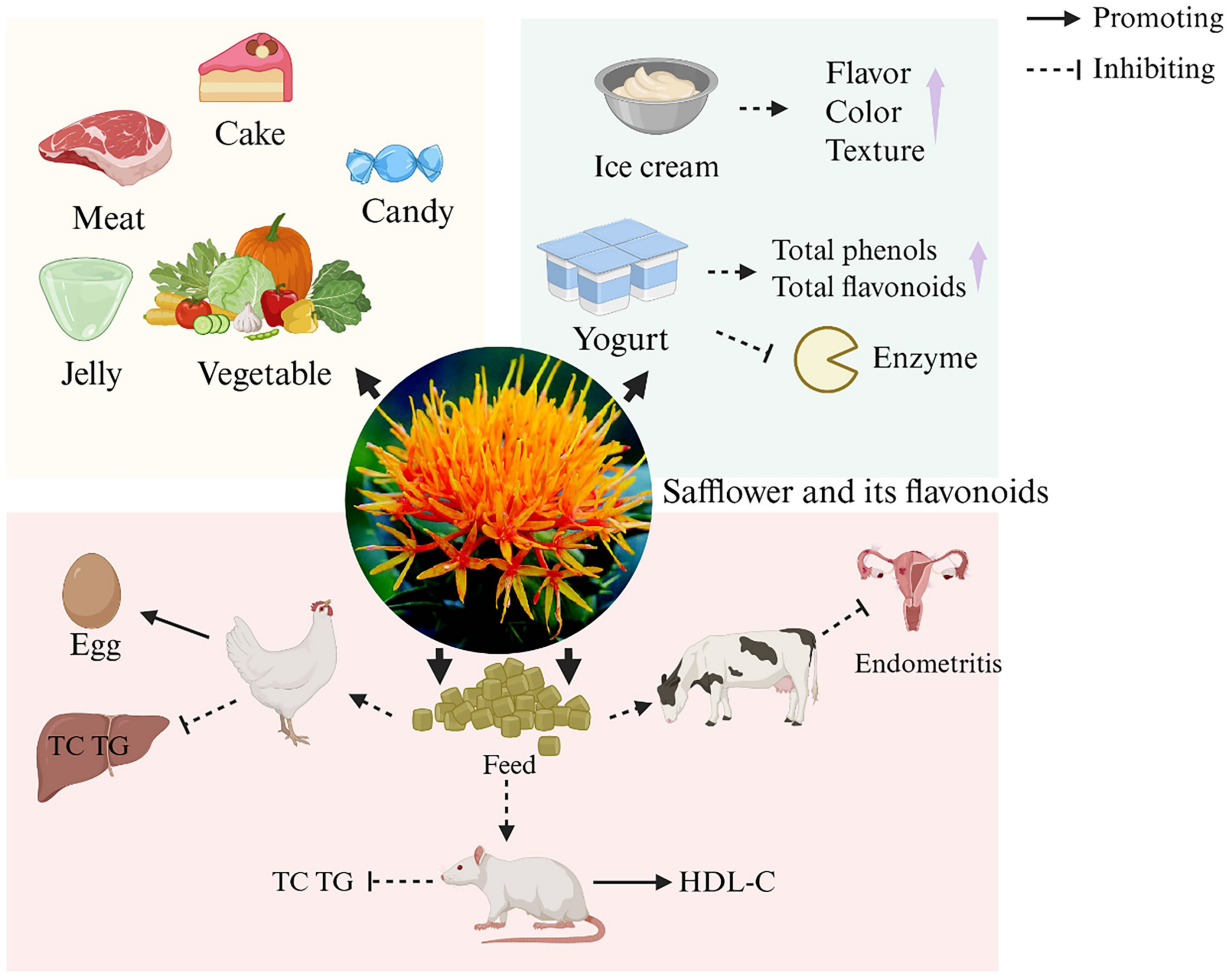
The application of safflower and its flavonoids in food and feed. (HDL-C, high density lipoprotein cholesterol; TC, total cholesterol; TG, triglyceride). Created in BioRender. Nû, SÑ. (2025) https://BioRender.com/8hi6l5f.

At the same time, SY pigment added to the feed of different animals has many positive effects in animal production applications. SY pigment enhances egg production in laying hens and boosts growth in piglets when added to their feed. An et al. (117) indicates that incorporating crude SE into daily poultry feed can significantly enhance the egg production rate and daily egg yield of laying hens without affecting egg weight. Regarding lipid metabolism regulation, it reduces hepatic total cholesterol and triglyceride levels while elevating hepatic 3-hydroxy-3-methylglutaryl-coenzyme A reductase activity. This intervention increases neutral sterol excretion, thereby aiding in serum cholesterol reduction. Furthermore, it demonstrates no adverse effects on yolk nutritional components (total cholesterol, triglycerides, etc.) or fatty acid composition. Research by Cho et al. (118) also revealed the potential of safflower as a feed additive. Supplementing rat feed with SE reduced plasma total cholesterol, triglycerides, and hepatic total cholesterol in ovariectomized rats while increasing plasma high density lipoprotein cholesterol levels. It improved blood lipid profiles by promoting fecal total cholesterol excretion, with no adverse effects on bile acid excretion. Furthermore, administering HSYA to dairy cattle helps prevent and manage endometritis. In summary, safflower flavonoids demonstrate significant application potential in food and feed industries. However, their primary active components exhibit high solubility in water but limited solubility in oil, coupled with poor gut mucosal permeability. These properties result in low oral bioavailability, thereby constraining their broader utilization in food and feed applications (119). Therefore, enhancing the bioavailability of safflower flavonoids in food and feed applications requires addressing their physicochemical solubility disparities and intestinal absorption barriers. Strategies such as nanoscale encapsulation (110), natural deep eutectic solvents (120) for stability optimization, and novel composite materials (119) to improve gut mucosal permeability can be employed. These approaches will fully unlock their bioactive potential, including antioxidant and metabolic regulatory functions, ultimately establishing a robust foundation for their scaled utilization in functional food development and feed additive formulations. The application of safflower and its flavonoids in food and feed is shown in Figure 7.

## 4 Nutraceutical importance of safflower

Safflower is highly significant in preventing and improving chronic diseases and maintaining human health due to its high biosafety and multiple health benefits. As a traditional medicinal and edible plant, safflower is rich in various nutrients crucial for human health. Safflower seeds contain an extremely high content of unsaturated fatty acids, with linoleic acid accounting for 70–80% (121). Linoleic acid, an essential unsaturated fatty acid for the human body, is a key component of cell membranes and participates in various physiological activities. Research by Meng et al. (122) demonstrated that safflower can enhance the immune function of carp by producing short-chain fatty acids and modulating the gut microbiota. Moreover, safflower is rich in flavonoids, which exhibit potent antioxidant activity. Studies indicate that safflower flavonoids can be incorporated as natural antioxidants into dairy products, cereal-based foods, and functional beverages. Beyond extending shelf life, they confer functional benefits such as modulating glucose-lipid metabolism and enhancing immune response (123, 124). Hong et al. (116) developed safflower yogurt by adding 0–1.0% safflower extract to plain yogurt. Their results showed that prolonged fermentation time led to significant dose-dependent enhancements in the yogurt’s free radical scavenging capacity, ROS suppression, and α-glucosidase inhibitory activity. The unique safflower pigments not only impart natural coloration to the plant, but also demonstrate vasodilatory effects and improve microcirculation (125, 126). Beyond these benefits, safflower demonstrates significant potential in neuroprotection, antitumor applications, and cosmetic formulations. Its well-documented safety profile provides a solid foundation for widespread use in nutritional supplements, functional foods, and clinical adjuvant therapies. This dual advantage of safety and efficacy positions safflower as a unique candidate for modern precision nutrition—particularly for developing low-dose, high-bioavailability dietary supplements targeting complex health conditions like metabolic syndrome and neurodegenerative diseases. In cosmetics, its natural pigments and antioxidant components offer exceptional safety for sensitive skin care products. As scientific research progresses, the multifaceted nutritional value of safflower continues to gain new scientific significance.

## 5 Discussion

Safflower, a pivotal herb in traditional medicine, demonstrates remarkable therapeutic efficacy in treating gynecological disorders and cardiovascular-cerebrovascular diseases. These pharmacological effects are primarily attributed to flavonoid compounds, particularly HSYA as the representative constituent. This review systematically summarizes the diverse biological activities of safflower flavonoids, including cardiocerebrovascular protection, antioxidant effects, and antitumor properties. Through comprehensive mechanistic investigations, we elucidate their multi-level biological effects across species, tissues, and molecular dimensions—encompassing signal transduction pathways, gene regulatory networks, protease inhibition, and growth cycle modulation. Furthermore, we highlight innovative applications in pharmaceutical development, functional foods, industrial dyes, and livestock production.

Notably, recent years have witnessed groundbreaking advances in the research of safflower bioactive compounds, particularly in elucidating biosynthetic pathways and discovering novel chemical constituents. Wang et al. (127) identified four key genes (CtF6H, CtCHI1, CtCGT, and Ct2OGD1) responsible for HSYA biosynthesis. Through substrate screening, they determined naringenin to be the crucial biosynthetic precursor of HSYA. Specifically, CtF6H converts naringenin to 6-hydroxynaringenin (taxifolin), while CtCHI1 catalyzes the conversion of taxifolin to safflomin chalcone. Ct2OGD1 cooperates with CtCGT to catalyze the di-C-glycosylation and dearomatization of safflomin chalcone, ultimately producing HSYA. Remarkably, these enzymes worked synergistically to achieve *de novo* HSYA synthesis in tobacco, establishing a novel pathway for green biosynthesis of HSYA. Xi et al. (128) identified a glycosyltransferase, UGT95A2, in safflower (*Carthamus tinctorius*) that significantly modulates the stability and pharmacological efficacy of safflower flavonoids by catalyzing 3′-hydroxyl glycosylation of the flavonoid B-ring. CtMYB76 was identified as a key regulatory factor for flavonoid biosynthesis in safflower. Heterologous overexpression of CtMYB76 in tobacco significantly upregulated the expression of structural genes involved in flavonoid production, leading to a substantial increase in flavonoid content. Further validation through dual-luciferase reporter assays and yeast one-hybrid experiments confirmed that CtMYB76 directly binds to and activates the promoters of key flavonoid biosynthetic genes (129), these findings suggest that future research could employ gene editing technologies to precisely modulate the expression of UGT95A2, CtMYB76, and related genes, thereby facilitating the development of novel safflower cultivars with enhanced flavonoid content. In the study of novel bioactive components in safflower, Zhang et al. (130) successfully isolated three highly oxidized quinone-chalcone C-glycoside rearrangement derivatives (safflospentosides A–C, compounds 1–3) from safflower yellow pigments. Significantly, compound 2 exhibited remarkable neuroprotective effects at 10 μM concentration, showing strong inhibitory activity against neuronal damage induced by both glutamate excitotoxicity and oxygen-glucose deprivation in rat cerebral cortex neurons. Liu et al. (131) isolated several novel compounds from safflower, including a new flavonoid (saffloflavanside, compounds 1), a new sesquiterpenoid (safflomegastigside, compounds 2), and a new amide derivative (saffloamide, compounds 3), along with 22 known compounds (compounds 4–25). MTT and immunofluorescence assays demonstrated that compounds 2–3, 8–11, and 15–19 exhibited protective effects against LPS-induced BEAS-2B cell injury. Furthermore, these compounds significantly suppressed nuclear translocation of p-p65. The discovery of these novel compounds highlights that the chemical diversity of safflower remains far from fully understood, and current research on its chemical constituents and bioactivities is still limited.

Beyond these findings, current research on safflower flavonoids still faces several critical unresolved issues. First, although safflower contains abundant flavonoid constituents with diverse pharmacological activities, existing studies have predominantly focused on HSYA, while research on other bioactive flavonoids remains substantially inadequate. Second, studies have reported that the antioxidant activity of safflower flavonoids is closely associated with their structural features (132). We therefore hypothesize that the difference in antioxidant activity between HSYA and SYA may be attributed to variations in the number and positioning of hydroxyl (–OH) groups. Structural modification of less potent flavonoid components in safflower based on these findings could significantly enhance their application efficiency. Third, as is well-documented, HSYA exhibits poor oral bioavailability (133), how can we improve the oral bioavailability, intestinal absorption, and *in vivo* stability of HSYA? Fourth, relevant studies have demonstrated that safflower extract exhibits significant negative effects on female reproductive hormones and adversely impacts ovarian development (134), although the tested concentrations of HSYA (single administration) show no significant effects on cell viability, the potential toxicity of long-term HSYA use requires systematic evaluation in future studies. Fifth, the angiogenic effect of HSYA makes it an ideal choice for treating various ischemic diseases, simultaneously, HSYA’s anti-angiogenic effects on neovascular diseases and cancer warrant equal attention. Further investigation is required to elucidate the underlying mechanisms of its bidirectional regulation of angiogenesis. Sixth, HSYB, as an isomer of HSYA, can be used for the treatment of breast cancer, while HSYA can be used for the treatment of liver cancer and lung cancer. Whether the efficacy can be enhanced when the two are used in combination deserves further exploration and consideration. Seventh, currently only the therapeutic effect of safflower flavonoids on non-infectious inflammation has been studied. Expanding the inhibitory effect of safflower flavonoids on bacterial infections might lead to a better development of safflower flavonoids. Eighth, currently, HSYA is mainly used in the treatment of cardiovascular and cerebrovascular diseases. Its potential in the respiratory system, liver, metabolic diseases, and malignant tumors should be further explored.

## 6 Conclusion

Safflower has a long history of medicinal use. Known for its effects of activating blood circulation and dredging meridians, as well as dissipating blood stasis and relieving pain, it is a commonly used blood-activating herb in clinical practice with broad application prospects. In this review, we discuss the protective effects of safflower flavonoids on the cardiovascular and cerebrovascular systems, along with their biological activities such as antioxidant, neuroprotective, antitumor, immunomodulatory, and anti-inflammatory properties. We also note that safflower flavonoids have been widely applied in multiple areas, including the protection of cardiovascular and cerebrovascular systems and the treatment of gynecological diseases. Furthermore, due to their extensive biological activities and high safety profile, they are gradually being utilized in the food industry and animal husbandry. However, in practical research and application, safflower flavonoids still face several pressing issues that need resolution. Further exploration and research are required on aspects such as bioavailability, potential toxicity, dosage optimization, in-depth studies on different organs and tissues, and the expansion of application fields, so that their potential can be further developed and utilized.

## Glossary

6-OHDA: 6-Hydroxydopamine
Aβ: Amyloid β-protein
ACS: Acute coronary syndrome
AD: Alzheimer’s disease
ADP: Adenosine diphosphate
AHSYB: Anhydrosafflor yellow B
AIF: Apoptosis inducing factor
Akt: Protein kinase B
ALI: Acute lung injury
AMPK: AMP-dependent protein kinase
AP-1: Activator protein-1
APOE: Apolipoprotein E
APP: Amyloid precursor protein
BACE1: Beta-secretase 1
BAX: Bcl-2 associated X protein
Bcl-2: B-cell lymphoma-2
BSCB: Blood-spinal cord barrier
CAT: Catalase
CDK2: Cyclin-dependent kinases 2
COPD: Chronic obstructive pulmonary disease
COX-2: Cyclooxygenase-2
CREB: CAMP-response element binding protein
CVD: Cardiovascular diseases
CypD: Cyclophilin D
cAMP: Cyclic adenosine monophosphate
DA: Dopamine
DCM: Diabetic cardiomyopathy
DHI: Dan Hong injection
DN: Diabetic nephropathy
DOX: Doxorubicin
EC: Endothelial cell
ECM: Extracellular matrix
EETs: Epoxyeicosatrienoic acids
eNOS: Endothelial nitric oxide synthase
ERK: Extracellular regulated protein kinases
ET: Endothelin
FOXP3: Forkhead box protein P3
GHI: Guhong injection
GPX4: Glutathione peroxidase 4
GSH: Glutathione
GSH-Px: Glutathione peroxidase
GSSG: Glutathione oxidized
GSK-3-β: Glycogen synthase kinase-3-β
HCC: Hepatocellular carcinoma
HO-1: Heme oxygenase-1
HSYA: Hydroxy safflor yellow A
HSYB: Hydroxy safflor yellow B
IBA1: Ionized calcium-binding adapter molecule 1
IκB: Inhibitor of NF-κB
IL: Interleukin
iNOS: Inducible nitric oxide synthase
I/R: Myocardial ischemia/reperfusion
JAK2: Janus kinase 2
JNK: c-Jun N-terminal kinase
LDL: Low-density lipoproteins
lncRNAs: Long non-coding ribonucleic acids
LPS: Lipopolysaccharide
MAP-2: Microtubule-associated protein-2
MAPK: Mitogen-activated protein kinase
MCAO: Middle cerebral artery occlusion
MCAO/R: Middle cerebral artery occlusion and reperfusion
MDA: Malondialdehyde
MEK: Mitogen-activated extracellular signal-regulated kinase
miRNAs: microRNAs
MI: Myocardial infarction
MMP: Mitochondrial membrane potential
mPTP: Mitochondrial permeability transition pore
MPO: Myeloperoxidase
mTOR: Mammalian target of rapamycin
MVD: Mevalonate pyrophosphate decarboxylase
NF-κB: Nuclear factor kappa-B
NLRP3: NOD-like receptor heat protein domain associated protein 3
NO: Nitric oxide
NOX4: NADPH oxidase 4
Nrf2: Nuclear factor erythroid 2-related factor 2
OGD/R: Oxygen-glucose deprivation/reoxygenation
PAF: Platelet activating factor
PAH: Pulmonary arterial hypertension
PAI: Plasminogen activator inhibitor
PAC-1: procaspase activating compound 1
PaCO_2_: CO_2_ partial pressure
PaO_2_: Oxygen partial pressure
PARP-1: Poly adenosine diphosphate-ribose polymerase-1
PCOS: Polycystic ovary syndrome
PCI: Percutaneous coronary intervention
PD: Parkinson’s disease
PGI2: Prostaglandin-I-2
PHZ: Phenyl hydrazine
PI3K: Phosphatidylinositol 3-kinase
PKA: Protein kinase A
PPARγ: Peroxisome proliferator-activated receptor-γ
PS1: Presenilins 1
PTEN: Phosphatase and tensin homolog
RORγt: Retinoic acid receptor-related orphan receptor gamma-t
ROS: Reactive oxygen species
SAFE: Safflower flavonoid extract
SCI: Spinal cord injury
SE: Safflower extract
SFI: Safflower injection
SIRT1: Silent information regulator 1
SLN: Solid lipid nanoparticles
sAPPα: Soluble amyloid protein precursor α
SOCS-3: Suppressor of cytokine signalling-3
SOD: Superoxide dismutase
STAT1: Signal transducer and activator of transcription 1
SY: Safflower yellow
SYA: Safflor yellow A
SYB: Safflor yellow B
SYI: Safflower yellow injection
TAB2: TGF-beta activated kinase 1 (map3k7) binding protein 2
TAK1: Transforming growth factor-β-activated kinase 1
TBI: Traumatic brain injury
T2DM: Type II diabetes mellitus
t-BOOH: Tert-butyl hydroperoxide
TH: Tyrosine hydroxylase
TLR4: Toll-like receptor 4
TNF-α: Tumor necrosis factor-α
TNFR1: TNF receptor type 1
t-PA: Tissue-type plasminogen activator
TRPV: Transient receptor potential vanilloid
TREM2: Triggering receptor expressed on myeloid cells 2
TSD: Taohong Siwu Decoction
TXA2: Thromboxane A2
VEGF: Vascular endothelial growth factor
WSB1: WD repeat and SOCS box containing 1
