# Supplementary material for: Pharmacological actions and applications of safflower flavonoids
Source: Front Nutr. 2025 Aug 6;12:1637053. doi: 10.3389/fnut.2025.1637053 (PMC12364913; doi:10.3389/fnut.2025.1637053)
Supplement: Supplementary file 1 [file Table_1.doc]

**Table 1 Invention patents of safflower flavonoids in cardiovascular and cerebrovascular diseases**

| Application Type | Pseudolatry acid | Base | Manufacturer | Functionality | Web address |
| --- | --- | --- | --- | --- | --- |
| Pharmaceuticals, healthcare products | A pharmaceutical composition for the treatment of cardiovascular diseases | Salvianolic acid B, hydroxy Safflower yellow pigment A | Chengdu PureFlex Pharmaceutical Research & Development Co. | Reduce thrombus size, inhibit thrombosis, reduce platelet coagulation rate, treat and prevent cardiovascular and cerebrovascular diseases | <http://pss-system.cnipa.gov.cn/sipopublicsearch/patentsearch/showViewList-jumpToView.shtml> |
| Pharmaceutical compositions and kits | A pharmaceutical composition and kit of hydroxy Safflower yellow pigment A and salvinorin A | Hydroxy Safflower yellow pigment A, salvinorin A | Zhejiang Yongning Pharmaceutical Co. | Treatment of cardiovascular diseases | <http://pss-system.cnipa.gov.cn/sipopublicsearch/patentsearch/showViewList-jumpToView.shtml> |
| Pharmaceuticals | A pharmaceutical composition containing astragalus polysaccharide and its pharmaceutical use | Astragalus polysaccharide or astragaloside, total phenolic acid of Salvia officinalis and/or Safflowerin (Safflowerin can be replaced by hydroxy Safflower yellow pigment A) | Xi'an Century Shengkang Pharmaceutical Co. | Treatment of kidney disease, diabetic nephropathy, cardiovascular disease or oncological disease | <http://pss-system.cnipa.gov.cn/sipopublicsearch/patentsearch/showViewList-jumpToView.shtml> |
| Pharmaceuticals | A kind of mulberry flower tablet and its preparation process and application in the treatment of cardiovascular and cerebrovascular diseases | Sangzhi, Safflower, Salvia miltiorrhiza, Rhizome Logistic Chuanxiong, Radix et Rhizome Dioscoreae, Astragalus | Ankang Central Hospital | Enhances hypoxia tolerance and improves platelet aggregation and adhesion | <http://pss-system.cnipa.gov.cn/sipopublicsearch/patentsearch/showViewList-jumpToView.shtml> |
| Pharmacy | Safflower yellow pigment and its application in the preparation of drugs for the treatment of cardiovascular diseases | Novel Safflower yellow pigments: 4 β D glucose 2,2,4,5 tetrahydroxy 6 [(2E) 3 (4 hydroxyphenyl) 1 ketone 2 propylidene 1] 5 cyclohexene 1,3 diketone (I 1), 2,4 dibeta D glucose 3,4,5 trihydroxy 6 [(2E) 3 (3,4 dihydroxyphenyl) 1 ketone 2 propylidene 1] 2,5 cyclohexadiene 1 ketone (I 2), 4 β D glucose 4,5 dihydroxy 6 [(2E) 3 (4 hydroxyphenyl) 1 ketone 2 propyl 1 group] 5 cyclohexene 1,3 dione (I 3) | Zhejiang Yongning Pharmaceutical Co. | Antioxidant, anti-platelet aggregation, treatment of cardiovascular diseases | <http://pss-system.cnipa.gov.cn/sipopublicsearch/patentsearch/showViewList-jumpToView.shtml> |
| Chinese medicine preparation | Danhong injection for cardiovascular disease | Salvia divinorum 10-100 parts, Safflower 0.33-3.3 parts, glucose 3.3-33.3 parts, sodium chloride 0.1-18 parts, 1000 parts of water | Yanmin Huang | Treatment of coronary heart disease, angina pectoris, myocarditis, ischemic encephalopathy, cerebral thrombosis, pediatric crying, gastric ulcer, anti-hepatic fibrosis | <http://pss-system.cnipa.gov.cn/sipopublicsearch/patentsearch/showViewList-jumpToView.shtml> |
| Technical area of Chinese medicine | A kind of traditional Chinese medicine for treating cardiovascular and cerebrovascular diseases and preparation method | Astragalus, Salvia miltiorrhiza, Radix et Rhizome Ginseng, Ophiopogonis, Zedoaria，Radix et Rhizoma Glycyrrhizae, Schisandra chinensis, Radix Rehmanniae Praeparatae, Radix Angelicae Sinensis, Safflower，Gui Zhi, Radix Paeoniae Alba, Rhizoma Ligustici Chuanxiong, Hawthorn, Poria, Rhizome Coptidis，Citrus aurantium dulcis, dragon bone, oyster, cassia seed, gibberellic acid, psyllium seed | Wankai Ma | Reducing blood viscosity, changing the state of myocardial tissue ischemia and hypoxia, and gradually restoring cardiac function | <http://pss-system.cnipa.gov.cn/sipopublicsearch/patentsearch/showViewList-jumpToView.shtml> |
| Oral sublingual spray field | A novel oral sublingual spray for dissolving thrombus and removing blood stasis | Safflower, Ice Tablet, Leech, Rhizome Ligustici Chuanxiong, Musk (Artificial), Salviae Miltiorrhizae, Centipede, Scorpion, Sandalwood, Gypsum, Pueraria Mirifica, Radix et Rhizome Glycyrrhizae, Panax notoginseng, Soapwort, Boswellia serrata | Sichuan Anzhenghui Pharmaceutical Technology Co. | Dissolve blood vessel blockage, prevent sudden death, prevent cerebral infarction, heart attack, sudden death, three highs, coronary heart disease, varicose veins | <http://pss-system.cnipa.gov.cn/sipopublicsearch/patentsearch/showViewList-jumpToView.shtml> |
| Chinese Medicine Preparation Field | A kind of Safflower injection and its preparation method | Hydroxy Safflower yellow pigment | Wuhan Fuxing Biopharmaceutical Co. | Prevention and treatment of occlusive cerebrovascular disease, coronary heart disease and vasculitis. | <http://pss-system.cnipa.gov.cn/sipopublicsearch/patentsearch/showViewList-jumpToView.shtml> |
| Technical area of Chinese medicine | A kind of Safflower injection enriched with hydroxy Safflower yellow pigment A and its preparation method | Hydroxy Safflower yellow pigment A | Lanzhi Group Wanrong Pharmaceutical Co. | Treatment of occlusive cerebrovascular disease, coronary heart disease, vasculitis, etc. | <http://pss-system.cnipa.gov.cn/sipopublicsearch/patentsearch/showViewList-jumpToView.shtml> |
| Technical area of Chinese medicine | A preparation method of Safflower injection | safflower (Carthamus tinctorius) | Hubei Wudang Jinding Pharmaceutical Co. | Treatment of cardiovascular disease | <http://pss-system.cnipa.gov.cn/sipopublicsearch/patentsearch/showViewList-jumpToView.shtml> |
| Traditional Chinese Medicine | A preparation method of Safflower injection | safflower (Carthamus tinctorius) | Jiangxi Tianshikang Traditional Chinese Medicine Co. | Treatment of cardiovascular diseases | <http://pss-system.cnipa.gov.cn/sipopublicsearch/patentsearch/showViewList-jumpToView.shtml> |
| Traditional Chinese Medicine Pharmaceutical Technology Area | A kind of nano Safflower yellow pigment injection and its preparation method | Safflower yellow pigment with appropriate amount of emulsifier and lipid co-emulsifier | Qinglong Zhang | Easily crosses the blood-brain barrier and has a slow-release effect | <http://pss-system.cnipa.gov.cn/sipopublicsearch/patentsearch/showViewList-jumpToView.shtml> |
| oral disintegrating tablet (ODT) | Safflower yellow pigment orally disintegrating tablets and its preparation process | Safflower yellow pigment, binder, filler, disintegrant, flavor modifier, coating material, effervescent agent, retention aid, lubricant | Beijing Kexinbicheng Pharmaceutical Technology Development Co. | Coronary artery dilation, antioxidant, myocardial protection, blood pressure lowering, immunosuppressive and cerebral protective effects | <http://pss-system.cnipa.gov.cn/sipopublicsearch/patentsearch/showViewList-jumpToView.shtml> |
| Drops and their preparation | Safflower yellow pigment drops and method of manufacture and application thereof | Safflower yellow pigment and at least one substrate selected from polyethylene glycol, gelatin, stearic acid, polyvinylpyrrolidone, glycerol monostearate | Zhejiang Yongning Pharmaceutical Factory | Treatment or prevention of a wide range of cardiovascular, cerebrovascular and other blood circulation disorders | <http://pss-system.cnipa.gov.cn/sipopublicsearch/patentsearch/showViewList-jumpToView.shtml> |
| Pharmaceutical chemistry field | New hydroxy Safflower yellow pigment pharmaceutical salt | safflower (Carthamus tinctorius) | Zhejiang Yongning Pharmaceutical Co. | Antiplatelet aggregation, coronary heart disease, angina pectoris, acute cerebral ischemia, etc. | <http://pss-system.cnipa.gov.cn/sipopublicsearch/patentsearch/showViewList-jumpToView.shtml> |
| Pharmaceutical preparations area | A compound preparation containing Safflower yellow pigment B and its application | Safflower yellow pigment B, resveratrol or resveratrol glycosides | Beijing Xinghao Pharmaceutical Co. | Protection of ischemic cardiomyocytes and ischemic brain cells from damage, which can be used in the preparation of drugs for the prevention and treatment of cardiovascular diseases | <http://pss-system.cnipa.gov.cn/sipopublicsearch/patentsearch/showViewList-jumpToView.shtml> |
| sublingual tablet (medicine) | A Safflower yellow pigment sublingual tablet and its differentiation method and application | One part Safflower yellow pigment, 3-4 parts filler and 1-2 parts disintegrant | Zhejiang Yongning Pharmaceutical Co. | Treatment of ischemic brain damage | <http://pss-system.cnipa.gov.cn/sipopublicsearch/patentsearch/showViewList-jumpToView.shtml> |
| Natural Medicinal Chemistry | Hydroxy Safflower yellow pigment A sodium and its production method and use | safflower (Carthamus tinctorius) | Zhejiang Yongning Pharmaceutical Co. | Antiplatelet aggregation, coronary heart disease, angina pectoris, acute cerebral ischemia and many other blood circulation disorders | <http://pss-system.cnipa.gov.cn/sipopublicsearch/patentsearch/showViewList-jumpToView.shtml> |
| Chinese Medicine Formulation Technology Field | An herbal formula for the treatment of cerebral hemorrhage in cardiovascular and cerebrovascular incompetence | Panax ginseng, Salvia miltiorrhiza, Tianma, Hawthorn, Radix Angelicae Sinensis, Rhizome Ligustici Chuanxiongg, Safflower, Paeonia lactiflora, Chinese Yam | Guizhou Baili Dujuan Qite Dream Food Development Co. | Anticoagulation, inhibit platelet aggregation, increase fibrinolytic activity, reduce blood viscosity, relieve vascular smooth muscle spasm, able to dilate blood vessels | <http://pss-system.cnipa.gov.cn/sipopublicsearch/patentsearch/showViewList-jumpToView.shtml> |
