# Supplementary material for: Pharmacological actions and applications of safflower flavonoids
Source: Front Nutr. 2025 Aug 6;12:1637053. doi: 10.3389/fnut.2025.1637053 (PMC12364913; doi:10.3389/fnut.2025.1637053)
Supplement: Supplementary file 2 [file Table_2.doc]

Table 2 Invention patents of safflower flavonoids in gynecological diseases

| Application Type | Pseudolatry acid | Base | Manufacturer | Functionality | Web address |
| --- | --- | --- | --- | --- | --- |
| Women's hygiene cloths | A kind of medicated cloth for women's hygiene with antibacterial and deodorising effect and its preparation method | Safflower Extract, Bitter Ginseng Extract, Peppermint Extract, Chai Beard Extract, Bamboo Leaf Extract, Lacmoid Extract, Chitosan Nanofibre, Tonic | Shanghai Yue Yue Shu Women's Products Co. | Strengthening the antibacterial and antimicrobial properties and deodorising properties of the cloth makes the sanitary products clean and hygienic, and avoids sanitary product infections. | <http://pss-system.cnipa.gov.cn/sipopublicsearch/patentsearch/showViewList-jumpToView.shtml> |
| Chinese Medicine Oral Liquid | An oral solution for the treatment of irregular menstruation with low volume and menopause | Radix Rehmanniae Praeparata, Radix Achyranthis Bidentatae, Radix Safflower, Rhizome Dioscoreae, Radix et Rhizome Glycyrrhizae, Radix Angelicae Sinensis, Rhizome Bacopa Monnieri, Rhizome Curcumae, Rhizome Ziziphi, Peach kernel, taiko ginseng, wolfberry, danshen, balsam. | Sichuan He's Biotechnology Co. | Effective treatment of irregular menstruation, oligomenorrhoea and menopause in women. | <http://pss-system.cnipa.gov.cn/sipopublicsearch/patentsearch/showViewList-jumpToView.shtml> |
| Chinese medicine technology | A drug and preparation method for treating infertility caused by female tubal incompatibility | Safflower, Sanguisorbia, Curcuma longa, leech, lutong, Chai Hu, Xiang Fu, Qing Pi, Angelica sinensis, Chinese yam, Bai Yao, Xia Ku Cao, Dandelion, Epimedium, Peach kernel, Citrus aurantium, Chicken-blooded vine. | Juan Dong | Removing blood stasis and clearing collaterals, promoting Qi and activating Blood are the mainstays, supplemented by promoting Qi and warming menstruation to disperse cold. | <http://pss-system.cnipa.gov.cn/sipopublicsearch/patentsearch/showViewList-jumpToView.shtml> |
| topical medicine | A formula for the treatment of gynecological diseases | Safflower, Millennium Health, Turbinaria, Angelica dahurica, Folium Artemisiae argyi, Radix Aesculi, Fructus Psoraleae, Wilsoniae, Xu Chang Qing, Chicken Blood Vine, Lu Tong, Green Chen Pi, Leeches, San Leng, Curcuma longa and Gui Zhi. | Jie Chen | Dispelling wind and dampness, warming the menstruation, dispersing cold, invigorating blood circulation, eliminating obstructions and dispersing lumps. | <http://pss-system.cnipa.gov.cn/sipopublicsearch/patentsearch/showViewList-jumpToView.shtml> |
| antiseptic | A formula and production method of gynecological herbal pads | Bitter ginseng, cypress, snakebite, safflower, peppermint, motherwort, haematoxylin, platycodon, ice tablet, mugwort, centella asiatica, dicotyledonin, earthworm bark. | Xi'an Haoran Pharmaceutical Co. | Anti-itching, antiseptic, anti-inflammatory and de-worming. | <http://pss-system.cnipa.gov.cn/sipopublicsearch/patentsearch/showViewList-jumpToView.shtml> |
| Chinese Medicine Preparation | An herbal composition for gynecological diseases | Bitter ginseng, snakeroot, safflower, atractylodes, aloe vera, angelica sinensis, goldenseal root, neem, ice chips, menthol. | Shaanxi Shiyu Pharmaceutical Co. | Clearing heat, drying dampness, invigorating blood and relieving menstrual cramps, improving menstrual disorders, vaginitis, cervicitis, pelvic inflammatory disease and gynecological tumours. | <http://pss-system.cnipa.gov.cn/sipopublicsearch/patentsearch/showViewList-jumpToView.shtml> |
| Chinese medicine technology | An herbal composition for alleviating and/or treating diseases of the female reproductive system | Safflower, angelica, peach kernel, chuanxiong rhizome, red herbs, danshen, sanlang, curcuma, frankincense, myrrh. | Xianghe County People's Hospital | Relieves the patient of abdominal cramps, pain, and increased vaginal discharge. | <http://pss-system.cnipa.gov.cn/sipopublicsearch/patentsearch/showViewList-jumpToView.shtml> |
